# Deficiency of ASGR1 in pigs recapitulates reduced risk factor for cardiovascular disease in humans

**DOI:** 10.1371/journal.pgen.1009891

**Published:** 2021-11-11

**Authors:** Baocai Xie, Xiaochen Shi, Yan Li, Bo Xia, Jia Zhou, Minjie Du, Xiangyang Xing, Liang Bai, Enqi Liu, Fernando Alvarez, Long Jin, Shaoping Deng, Grant A. Mitchell, Dengke Pan, Mingzhou Li, Jiangwei Wu

**Affiliations:** 1 Key Laboratory of Animal Genetics, Breeding and Reproduction of Shaanxi Province, College of Animal Science and Technology, Northwest A&F University, Yangling, Shaanxi, China; 2 Institute of Animal Genetics and Breeding, College of Animal Science and Technology, Sichuan Agricultural University, Chengdu, Sichuan, China; 3 Clinical Immunology Translational Medicine Key Laboratory of Sichuan Province, Sichuan Academy of Medical Sciences & Sichuan Provincial People’s Hospital, Chengdu, Sichuan, China; 4 Chengdu Clonorgan Biotechnology Co. LTD, Chengdu, Sichuan, China; 5 Institute of Cardiovascular Sciences, Health Science Center, Xi’an Jiao Tong University, Xi’an, Shaanxi, China; 6 Divisions of Gastroenterology, Hepatology and Nurition, University of Montreal and Centre Hospitalier Universitaire Sainte-Justine, Montreal, Quebec, Canada; 7 Divisions of Medical Genetics, Department of Pediatrics, University of Montreal and Centre Hospitalier Universitaire Sainte-Justine, Montreal, Quebec, Canada; HudsonAlpha Institute for Biotechnology, UNITED STATES

## Abstract

Genetic variants in the asialoglycoprotein receptor 1 (*ASGR1*) are associated with a reduced risk of cardiovascular disease (CVD) in humans. However, the underlying molecular mechanism remains elusive. Given the cardiovascular similarities between pigs and humans, we generated ASGR1-deficient pigs using the CRISPR/Cas9 system. These pigs show age-dependent low levels of non-HDL-C under standard diet. When received an atherogenic diet for 6 months, ASGR1-deficient pigs show lower levels of non-HDL-C and less atherosclerotic lesions than that of controls. Furthermore, by analysis of hepatic transcriptome and *in vivo* cholesterol metabolism, we show that ASGR1 deficiency reduces hepatic *de novo* cholesterol synthesis by downregulating 3-hydroxy-3-methylglutaryl-CoA reductase (*HMGCR*), and increases cholesterol clearance by upregulating the hepatic low-density lipoprotein receptor (*LDLR*), which together contribute to the low levels of non-HDL-C. Despite the cardioprotective effect, we unexpectedly observed mild to moderate hepatic injury in ASGR1-deficient pigs, which has not been documented in humans with *ASGR1* variants. Thus, targeting *ASGR1* might be an effective strategy to reduce hypercholesterolemia and atherosclerosis, whereas further clinical evidence is required to assess its hepatic impact.

## Author summary

Previous studies have reported an association between *ASGR1* variants and CVD in humans. However, the underlying mechanism is unknown. We used ASGR1-deficient pig to recapitulate the reduced risk features of CVD in humans with *ASGR1* variants, indicating that *ASGR1* inhibition could be an effective strategy to treat atherosclerotic CVD. Our results highlight the demand for taking advantage of genetically modified large animal models to investigate the pathogenesis and therapeutic development of CVD in humans. Unexpectedly, we demonstrate the first link between ASGR1 deficiency and liver injury, a feature that has not been documented in humans with *ASGR1* variants. These results suggest that *ASGR1* might be an effective target for reducing CVD, whereas revealing a genetic predisposition to liver disease in humans with *ASGR1* variants.

## Introduction

Cardiovascular disease (CVD) is the leading cause of morbidity and mortality worldwide with a major risk factor of elevated plasma levels of non-high-density lipoprotein cholesterol (non-HDL-C) [1]. Levels of non-HDL-C are influenced by environmental [2] and genetic factors [3]. Mutations in several genes including autosomal recessive hypercholesterolemia [4], 3-hydroxy-3-methylglutaryl-CoA reductase (HMGCR) [5], low-density lipoprotein receptor (LDLR) [6], LIM domain and actin-binding 1 (*LIMA1*) [7], Niemann-Pick C1-Like 1 (*NPC1L1*) [8] and proprotein convertase subtilisin/kexin type 9 (*PCSK9*) [9] affect non-HDL-C levels. *HMGCR*, *NPC1L1* and *PCSK9* are well-established clinical targets for hypercholesterolemia [8–11]. Nonetheless, mutations in these genes, despite conferring high risk, are rare and explain only a small portion of familial clustering of hypercholesterolemia [12], implying that other genetic factors remain to be identified [13].

The asialoglycoprotein receptor 1 (*ASGR1*) gene encodes the major subunit of the asialoglycoprotein receptor (ASGPR), a lectin receptor that is reported to recognize and mediate the endocytosis and degradation of many circulating desialylated glycoproteins [14]. Two loss-of-function variants in *ASGR1* (12 bp deletion in the fourth intron that causes a frameshift mutation and a premature stop codon [carried by 1 in 120 persons], and a 4 bp insertion that introduces a stop codon at position 158 in the protein [carried by 1 in 1850 persons]) were identified in an Iceland population, and *ASGR1* haploinsufficiency was associated with reduced levels of non-HDL-C and a reduced risk of coronary artery disease (CAD) [15]. However, the mechanisms underlying this association remain unclear. To this end, an appropriate animal model is required.

Pigs have emerged as a relevant biomedical model for dissecting complex diseases due to their anatomical, genetic and physiological similarities with humans [16,17]. Bama miniature pigs are one of the commonly used breeds for CVD study [18]. Thus, in this study, we created ASGR1-deficient pigs to investigate the mechanisms underlying the cardioprotective role of *ASGR1* variants in humans. The plasma lipoprotein profiles, as well as the atherosclerotic lesions, which are the most common indicators of CVD, were measured longitudinally in these animals under both a normal diet and a high-fat and high-cholesterol (HFHC) diet. As in humans with *ASGR1* variants [15], ASGR1 deficiency in pigs substantially reduces circulating non-HDL-C levels and thus protects against HFHC diet-induced atherosclerosis. Mechanistic studies reveal that the cholesterol-lowering effect of ASGR1 deficiency is achieved by reducing *de novo* cholesterol synthesis in liver through downregulation of HMGCR and enhancing LDL clearance by upregulation of the hepatic LDLR. Our findings indicate that ASGR1 inhibition has the potential for reducing both circulating non-HDL-C levels and development of atherosclerotic CVD.

## Results

### Generation of ASGR1 knockout pigs

**Fig 1:**
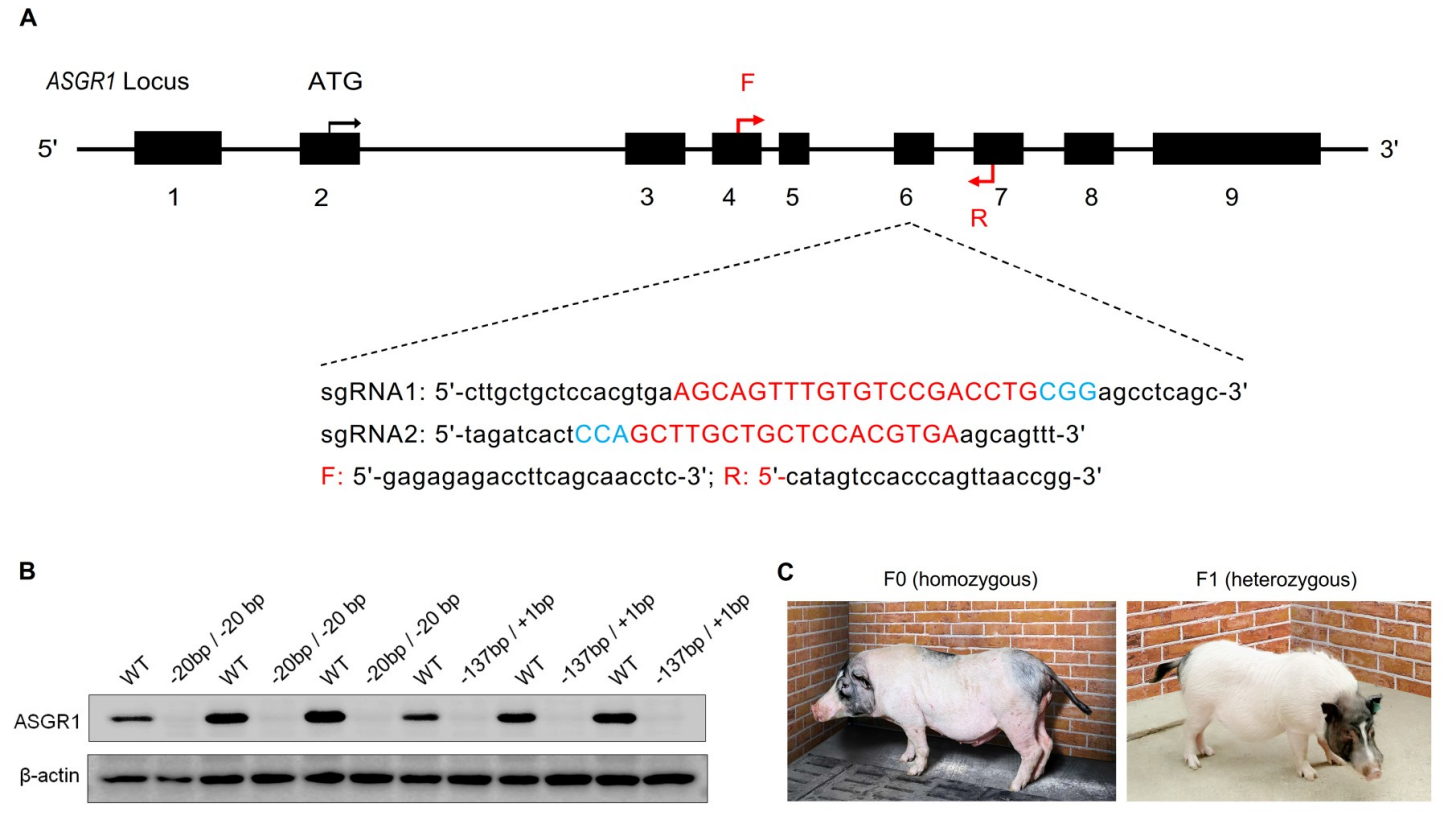
Generation of *ASGR1* knockout pigs. **A**. Schematic diagram of Cas9-sgRNA targeting sites of the pig *ASGR1* locus. Two sgRNAs were used to target pig *ASGR1* exon 6 to promote DNA breaks and homologous recombination. The sgRNA targeting sequences are shown in red, and the PAM sequences are shown in blue and are underlined. E1-E9: Exon 1–9; the black arrow: Site of the start codon; the red arrow: primer site of the genotyping. **B**. Western blots of ASGR1 and internal control β-actin in the livers of 24-month-old *ASGR1*^-/-^ pigs (the -20 bp / -20 bp and the -137 bp / +1 bp genotypes, *n* = 3 in each phenotype) and age-matched WT pigs (*n* = 6). **C**. Representative photograph of founder (*ASGR1*^-/-^) pigs and F1 (*ASGR1*^+/-^) pigs. PAM, protospacer adjacent motif; WT, wild-type.

To disrupt the *ASGR1* gene in Bama miniature pigs, we designed two candidate sgRNAs for CRISPR/Cas9 targeting (**Fig 1A**). In pig fibroblasts, sgRNA1 produced the expected insertions/deletions (Indels) in the targeting region of the *ASGR1* gene (9 of 40, or 22% of colonies), sgRNA2 produced no detectable indels and was not studied further. Transfection of the Cas9-sgRNA1 targeting vector into fibroblasts produced a total of 18 positive colonies, of which 15 carried biallelic modifications and the other three had monoallelic modifications. Five biallelic-mutation types were identified in the target region of *ASGR1* (**S1 Table**). Three biallelic-modified colonies, A1 (-20 bp / -20 bp), A2 (-137 bp / +1 bp) and A3 (+1 bp / +1 bp), were selected and used as donor cells for somatic cell nuclear transfer. Two pregnant recipient sows received the transfer of a total of 361 reconstructed embryos, and six live-born male piglets were naturally delivered (**S2 Table**). Genotyping by Sanger sequencing identified that all piglets were *ASGR1*^*-/-*^ (homozygous) pigs (**S1 Fig**). Three of the piglets (281, 330 and 332) were homozygous for the -20 bp mutation, corresponding to A1 donor cells and three (331, 333 and 334) had a -137 bp / +1 bp genotype, identical to A2 donor cells. As predicted, ASGR1 protein was undetectable in the liver of the *ASGR1*^*-/-*^ pigs compared to high levels in age-matched wild-type (WT) Bama miniature pigs (**Fig 1B**). We subsequently obtained 15 F1 pigs in **S3 Table**. Off-target effects were checked by PCR amplification and Sanger sequencing at the potential sites. No potential off-targets were shown in the F0 (homozygous, *ASGR1*^*-/-*^) and F1 (heterozygous, *ASGR1*^*+/-*^) generations of ASGR1-deficient pigs (**S4 and S5 Tables**). In this study, the characterization has focused on the F0 and F1 pigs (**Fig 1C**). The appearance and behavior of ASGR1-deficient pigs did not show obvious abnormalities.

### ASGR1-deficient pigs show age-dependent low levels of non-HDL-C

**Fig 2:**
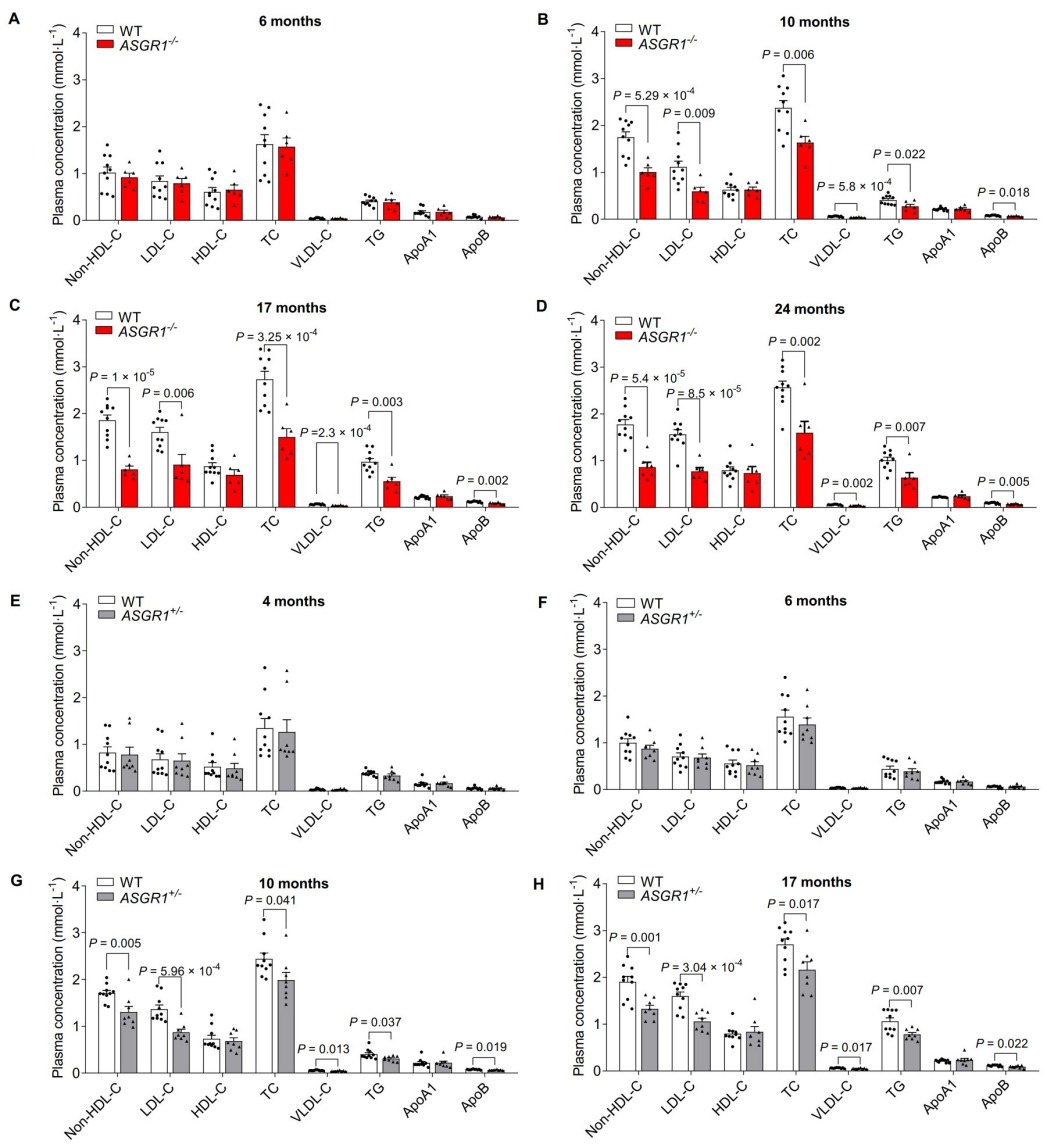
Plasma lipoprotein profiles of pigs under standard diet. **A-D**. The effect of age on levels of non-HDL-C, LDL-C, HDL-C, TC, VLDL-C TG, ApoA1 and ApoB in *ASGR1*^-/-^ pigs and WT controls receiving a standard diet (*n* = 6 for *ASGR1*^*-/-*^ pigs, *n* = 10 for WT controls. Data represent two independent experiments combined). **E-H**. The effect of age on levels of non-HDL-C, LDL-C, HDL-C, TC, VLDL-C TG, ApoA1 and ApoB in *ASGR1*^*+/-*^ pigs and WT controls receiving a standard diet. Points indicate data from individual pigs (*n* = 8 for *ASGR1*^*+/-*^ pigs, *n* = 10 for WT controls. Data represent two independent experiments combined). *P* values are shown for indicated comparisons by the Student’s *t*-test. Values are mean ± SEM. The underlying data for this figure can be found in **S1 Data**. ApoA1, apolipoprotein A1; ApoB, apolipoprotein B; HDL-C, high-density lipoprotein cholesterol; LDL-C, low-density lipoprotein cholesterol; non-HDL-C, non-high-density lipoprotein cholesterol; TC, total cholesterol; TG, triglycerides; WT, wild-type.

To investigate the roles of ASGR1 in the development of CVD, we longitudinally measured the plasma lipid profiles including high-density lipoprotein cholesterol (HDL-C), low-density lipoprotein cholesterol (LDL-C), total cholesterol (TC), very low-density lipoprotein cholesterol (VLDL-C), triglycerides (TG), apolipoprotein A1 (ApoA1) and apolipoprotein B (ApoB) in ASGR1-deficient pigs and WT controls fed a normal diet. At the age of six months, *ASGR1*^*-/-*^ founders showed levels of the aforementioned blood lipids comparable to age-matched WT Bama miniature pigs (**Fig 2A**). Nonetheless, by 10 months, lower levels of atherogenic factors were shown in *ASGR1*^-/-^ pigs than in controls: LDL-C (46.93%), TC (31.19%), VLDL-C (34.06%), non-HDL-C (42.51%), TG (32.43%) and ApoB (17.72%) (**Fig 2B**). Levels of the atheroprotective HDL-C and ApoA1 were similar in *ASGR1*^*-/-*^ and WT pigs. Similar patterns were observed at the ages of 17 and 24 months (**Fig 2C and 2D**). Since the reported loss-of-function *ASGR1* variants in humans are heterozygous, we next analyzed the changes in F1 generation (*ASGR1*^*+/-*^) pigs. We found comparable lipid levels in *ASGR1*^*+/-*^ pigs and WT controls at four and six months of age (**Fig 2E and 2F**), but at 10 months, *ASGR1*^*+/-*^ pigs showed markedly lower levels of LDL-C (36.27%), TC (18.58%), VLDL-C (21.98%), non-HDL-C (23.64%), TG (23.39%) and ApoB (14.64%) than that in WT pigs, without pronounced effects on levels of HDL-C and ApoA1 (**Fig 2G**). A similar pattern was observed at 17 months for *ASGR1*^*+/-*^ pigs (**Fig 2H**). Of note, changes in lipoprotein profiles for *ASGR1* heterozygous deficient pigs were similar to that of *ASGR1* heterozygous carriers in humans. Together, these results indicate that ASGR1 deficiency causes age-dependent lower levels of non-HDL-C and TG in pigs, recapitulating the lipoprotein profiles of humans with *ASGR1* variants.

### Deficiency of ASGR1 in pigs protects against HFHC diet-induced hypercholesterolemia and atherosclerotic lesions

**Fig 3:**
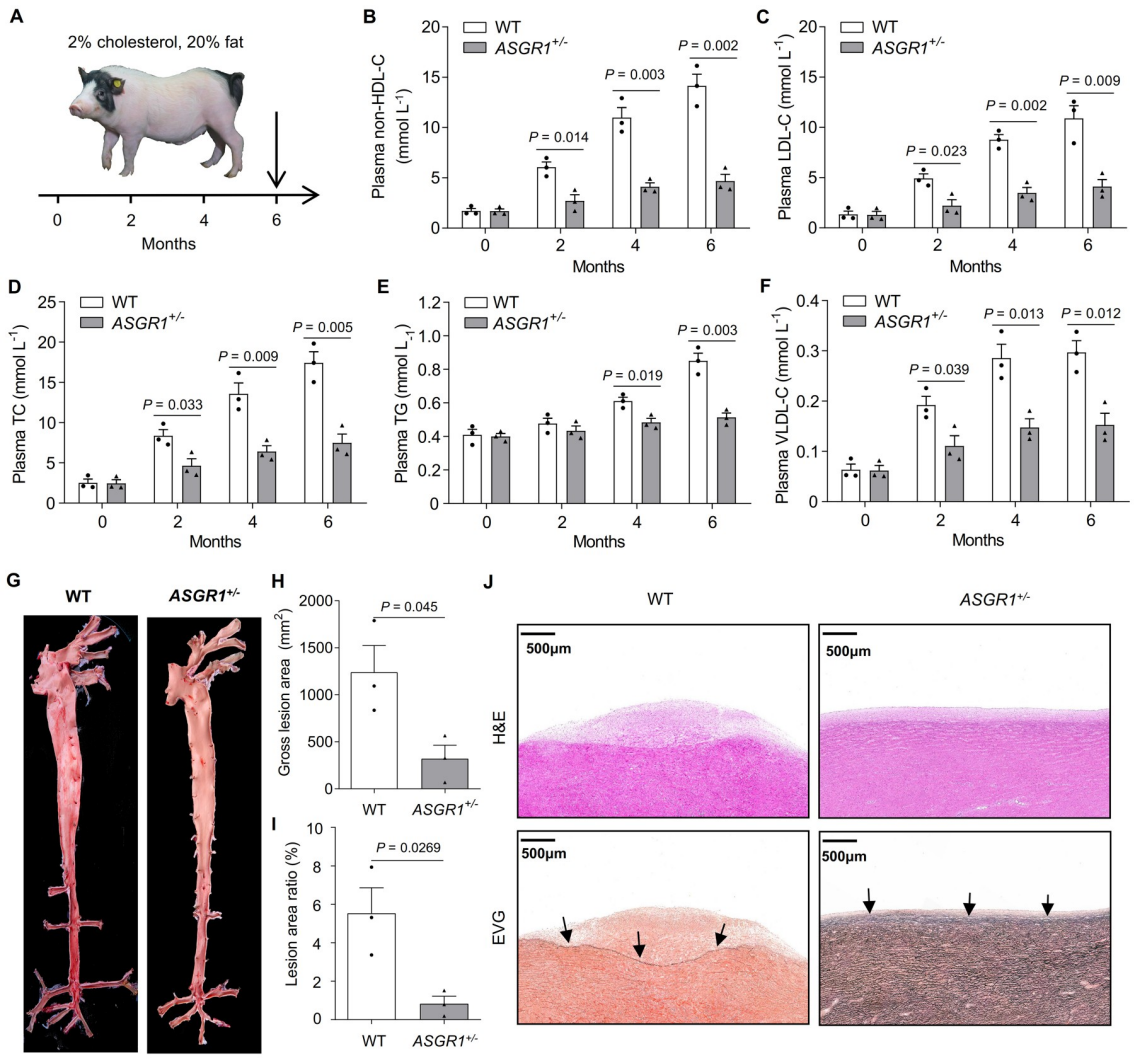
*ASGR1*^*+/-*^ pigs are protected from HFHC diet-induced hypercholesterolemia and atherosclerosis. Six-month-old *ASGR1*^*+/-*^ pigs and age-matched WT pigs were fed an HFHC diet for six months. **A.** Schematic diagram of pig treatment. **B-F.** Plasma levels of (**B**) non-HDL-C, (**C**) LDL-C, (**D**) TC, (**E**) TG and (**F**) VLDL-C at the indicated time points (*n* = 3 per group). **G.** Representative pictures of Sudan-IV-stained aortas showing atherosclerotic lesions in WT pigs but not in *ASGR1*^*+/-*^ pigs. **H, I.** (**H**) The gross lesion area and (**I**) the percentage of the Sudan-IV-stained area were measured by Image J software (*n* = 3 per group). **J.** Representative histological images of aortas from the aortic arch of WT and *ASGR1*^*+/-*^ pigs. Serial cuts from paraffin sections were stained with H&E and EVG, the black arrows point to the internal elastic lamina. *P* values are shown for indicated comparisons by the Student’s *t*-test. Values are mean ± SEM. The underlying data for this figure can be found in **S1 Data**. EVG, elastic van Gieson; H&E, hematoxylin-eosin; HFHC, high-fat and high-cholesterol; LDL-C, low-density lipoprotein cholesterol; non-HDL-C, non-high-density lipoprotein cholesterol; TC, total cholesterol; TG, triglycerides; VLDL-C, very-low-density lipoprotein cholesterol; WT, wild-type.

To examine whether the substantial reduction of atherogenic non-HDL-C upon ASGR1 deficiency would protect pigs against atherosclerosis, we assessed atherosclerotic lesions in two-year-old *ASGR1*^*-/-*^ pigs and their age-matched WT pigs fed a normal diet. By Sudan IV Staining of the aorta, neither *ASGR1*^*-/-*^ pigs nor WT controls showed atherosclerotic lesions (**S2A Fig**), suggesting that pigs do not easily develop atherosclerosis on a standard diet. To accelerate the process of atherosclerosis, *ASGR1*^*+/-*^ pigs and age-matched WT controls were challenged with a HFHC diet containing 20% saturated fat and 2% cholesterol [19], administered from the age of 6 months to 12 months (**Fig 3A**). Compared to age-matched pigs fed a normal diet (**Fig 2E and 2F**), HFHC diet feeding markedly increased levels of non-HDL-C, LDL-C, TC, TG, and VLDL-C in both *ASGR1*^+/-^ pigs and WT controls after two, four, and six months (**Fig 3B–3F**). Nonetheless, the increases were much smaller in *ASGR1*^*+/-*^ pigs than in WT controls. After six months of HFHC feeding, WT pigs exhibited an 8.25-fold increase in non-HDL-C levels, whereas only a 2.77-fold increase was observed in *ASGR1*^*+/-*^ pigs (**Fig 3B**). These results suggest that ASGR1-deficiency in pigs protects against HFHC diet-induced hypercholesterolemia. We used Sudan IV staining to assess the occurrence of atherosclerotic lesions in these pigs after six months of HFHC-diet feeding. At sacrifice (at the age of 12 months), gross examination and histopathological assessment revealed substantial areas of fatty streaks in the aorta of WT pigs, which is hardly detectable in the aorta of *ASGR1*^*+/-*^ pigs (**Figs 3G–3I and S2B**). Consistent with this, hematoxylin-eosin (H&E) and elastic van Gieson (EVG) staining showed smaller intimal lesion sizes in *ASGR1*^*+/-*^ pigs than in WT controls (**Fig 3J**). Besides, the numbers of macrophages in plaques were significantly reduced in *ASGR1*^*+/-*^ pigs under HFHC diet compared with age-matched WT pigs (**S3A–S3C Fig**). Together, these results show that ASGR1 deficiency protects against HFHC-induced hypercholesterolemia and atherosclerosis in pigs.

### ASGR1 deficiency in pigs decreases HMGCR-catalyzed hepatic cholesterol biosynthesis and increases hepatic LDLR-mediated LDL clearance

The cholesterol-lowering effect upon ASGR1 deficiency could be attributed to several factors such as reduction of intestinal absorption, *de novo* hepatic cholesterol synthesis, VLDL secretion, or increase of clearance by tissue uptake, as well as obesity. Basic characterization of pigs showed no difference in food intake, body weight or body fat content between ASGR1-deficient pigs and their age-matched WT pigs under normal diet feeding (**S4A–S4C Figs**), indicating that the reduced plasma non-HDL-C levels in the absence of ASGR1 are independent of adiposity. Furthermore, we observed comparable fecal TC and TG contents in ASGR1-deficient and WT pigs (**S4D and S4E Fig**). In line with this, we showed similar protein levels of NPCL1L and LIMA1, two key regulators of intestinal cholesterol absorption A LIMA1 [7,8], in small intestine of ASGR1-deficient and WT pigs (**S4F Fig**). Therefore, differential intestinal absorption is unlikely to be the primary reason for the reduced non-HDL-C levels in ASGR1-deficient pigs.

**Fig 4:**
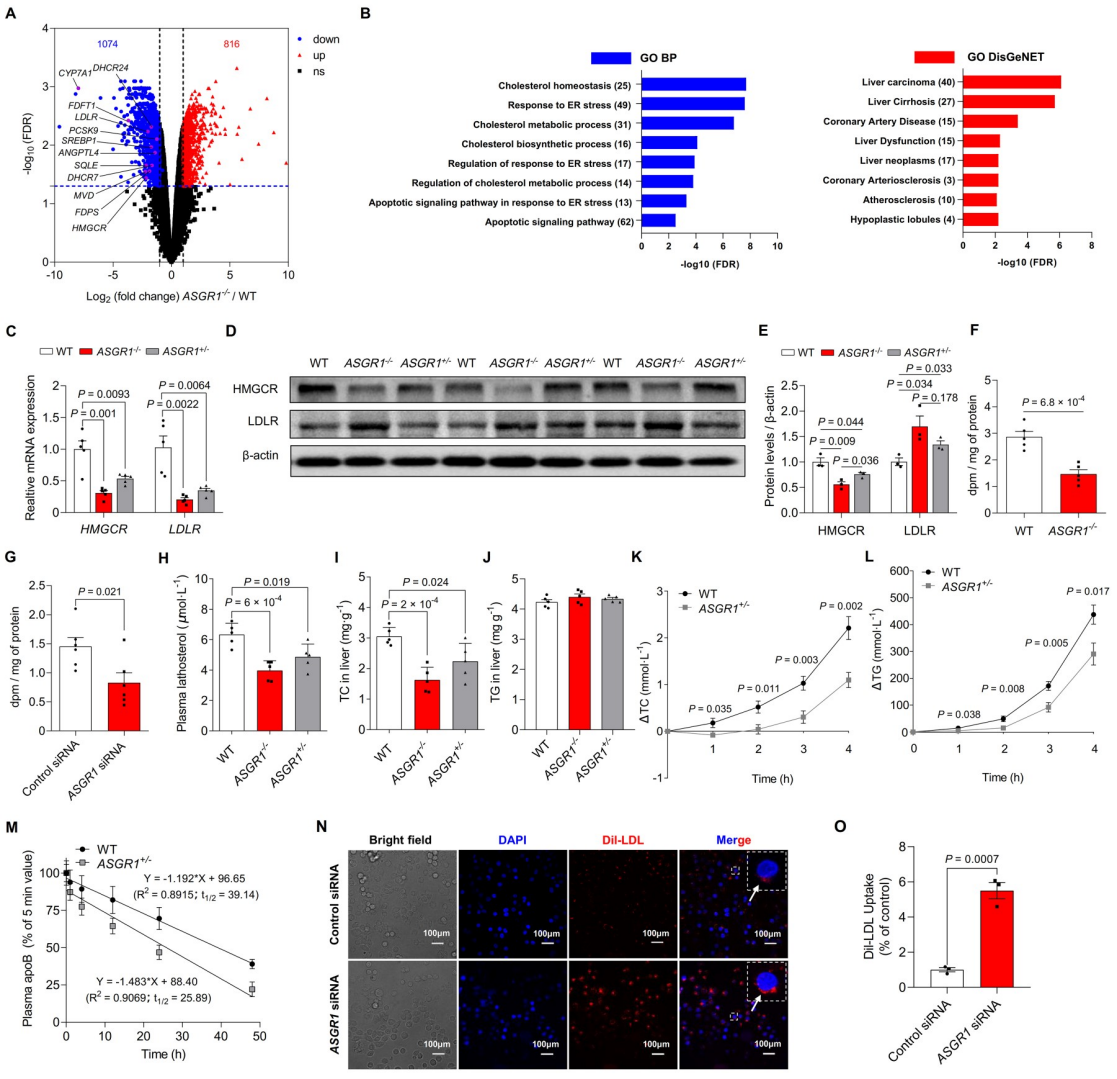
ASGR1 deficiency reduces hepatic cholesterol synthesis by downregulating HMGCR and augments cholesterol clearance by upregulating hepatic LDLR. **A.** Hepatic transcriptome profiling of *ASGR*^*-/-*^ pigs and WT controls. The volcano diagram shows the differentially expressed genes (fold change > 2 and FDR < 0.05; downregulated in blue and upregulated in red) upon ASGR1 deficiency, in which cholesterol metabolism-related genes were labeled in purple. The blue dashed line indicates the threshold for significance (FDR <0.05), and the black dashed lines indicate the threshold for fold change (fold change > 2). **B.** Top ranking functional terms enriched for differentially expressed genes using the Metascape tool. GO-BP: biological process (blue) and DisGeNET (red). **C.** Relative mRNA expression of *HMGCR* and *LDLR* in livers of 24-month-old WT and *ASGR1*^*-/-*^ pigs, points indicate data from individual pigs (*n* = 5 per group, data represent two independent experiments combined). **D, E.** (**D**) Representative western blotting and (**E**) quantification of HMGCR and LDLR in livers of pigs (*n* = 3 per group, data are representative of two independent experiments). **F.** Rate of cholesterol biosynthesis in hepatocytes from 24-month-old WT and *ASGR1*^*-/-*^ pigs (*n* = 5 per group, data represent two independent experiments combined). **G.** Representative rate of cholesterol biosynthesis in HepG2 cells with *ASGR1* knockdown by siRNA (*n* = 6 per group, data are representative of three independent experiments). **H.** Plasma lathosterol in ASGR1-deficient pigs and WT controls (*n* = 5 per group, data represent two independent experiments combined). **I, J.** (**I**) Liver total cholesterol (TC) and (**J**) total triglycerides (TG) content of pigs at 24 months (*n* = 5 per group, data represent two independent experiments combined). **K**, **L.** Changes in plasma (**K**) TC and (**L**) TG levels after Poloxamer-407 injection in pigs (*n* = 8 per group, data represent two independent experiments combined). **M.** Clearance of LDL-C in 12-month-old WT and *ASGR1*^*+/–*^pigs (*n* = 6 per group, data represent two independent experiments combined). **N, O.** (**N**) Representative confocal immunofluorescence microscopy images of Dil-LDL uptake in HepG2 cells transfected with si-control and si-*ASGR1* from three independent experiments. Confocal microscopic images represent the fluorescence intensity of Dil-LDL (red) and DAPI (blue). Scale bars, 100 μm. (**O**) Representative fluorescence quantification of isopropanol-extracted DiI-LDL (520 nm) (*n* = 3 per group, data are representative of three independent experiments). *P* values are shown. For analyses of intergroup differences between two groups, data were assessed using the Student’s *t*-test. For analysis of multiple comparisons, data were analyzed using ordinary one-way ANOVA followed by Tukey’s test. Values are mean ± SEM. The underlying data for this figure can be found in **S1 Data**. ANOVA, analysis of variance; DAPI, 4′, 6-diamidino-2-phenylindole; FDR, False Discovery Rate; HMGCR, 3-hydroxy-3-methylglutaryl-CoA reductase; LDL-C, low-density lipoprotein cholesterol; LDLR, low-density lipoprotein receptor; TC, total cholesterol; TG, triglycerides; WT, wild-type.

Given that the liver plays an important role in the regulation of cholesterol homeostasis [20], and is also the predominant site of ASGR1 expression [21], we hypothesized that the atheroprotective effect of ASGR1 deficiency may be mediated by the liver. We compared hepatic transcriptome profiling in the livers of 24-month-old *ASGR1*^*-/-*^ and WT pigs and identified expression changes in 1,890 genes (|fold change| > 2, *P* < 0.05, likelihood ratio test) (**Figs 4A and S5A and S5B**). This gene set was mainly enriched in functional terms related to the regulation of cholesterol metabolism and atherosclerosis (**Fig 4B**). Notably, we observed that 12 genes well-documented in relation to cholesterol metabolism were downregulated in livers of *ASGR1*^*-/-*^ pigs (**Fig 4A**). *HMGCR* is the rate-limiting enzyme for cholesterol *de novo* synthesis [22]. Validation of *HMGCR* expression in liver tissues confirmed markedly reduced mRNA (**Fig 4C**) and protein levels (**Fig 4D and 4E**) in ASGR1-deficient pigs. These results suggest that *ASGR1* may regulate hepatic cholesterol homeostasis by repressing *HMGCR*.

To test the effect of ASGR1 deficiency on hepatic cholesterol *de novo* synthesis *in vitro*, we measured the rate of cholesterol biosynthesis in the presence of tritium-labeled acetic acid in hepatocytes isolated from *ASGR1*^-/-^ pigs and WT controls, as well as in human HepG2 cells treated with *ASGR1* siRNA or control siRNA. ASGR1 deficiency reduced cholesterol biosynthesis rates in porcine hepatocytes and HepG2 cells compared to their respective controls (**Fig 4F and 4G**). Consistently, plasma levels of lathosterol, an indicator of whole-body cholesterol synthesis [23], were reduced in ASGR1-deficient pigs (**Fig 4H**). In agreement with these results, ASGR1-deficient pigs showed low levels of hepatic TC (**Fig 4I**), similar levels of hepatic TG (**Fig 4J**) and a low rate of VLDL secretion (**Fig 4K and 4L**). These results indicate that ASGR1 deficiency reduces hepatic cholesterol biosynthesis by repressing *HMGCR*, leading to decreased VLDL secretion and circulating LDL levels.

Reduced hepatic cholesterol synthesis is often associated coordinately with elevated LDLR-mediated hepatic clearance of circulating LDL [20,24,25]. The mRNA levels of *LDLR*, the classic regulator of cholesterol clearance, were reduced in livers of ASGR1-deficient pigs (**Fig 4C**). However, hepatic protein levels and immunofluorescence of LDLR were increased in ASGR1 deficiency (**Figs 4D and 4E and S6**). Next, we directly evaluated LDL clearance in pigs by intravenous injection of isolated pig LDL. *ASGR1*^*+/-*^ pigs showed a greater LDL clearance rate than WT controls, with a half-life of 25.89 h in *ASGR1*^*+/-*^ pigs versus 39.14 h in WT controls (**Fig 4M**). In line with this elevation *in vivo*, *ASGR1* knockdown increased LDL uptake in HepG2 cells (**Figs 4N and 4O and S7**). These results suggest that ASGR1 deficiency increases LDLR-mediated hepatic LDL uptake, which could contribute to the low levels of plasma non-HDL-C. Collectively, our results suggest that the reduced hepatic cholesterol *de novo* synthesis and elevated hepatic cholesterol uptake from circulation upon ASGR1 deficiency contribute to low levels of circulating non-HDL-C.

### ASGR1-deficient pigs show high circulating levels of liver enzymes

**Fig 5:**
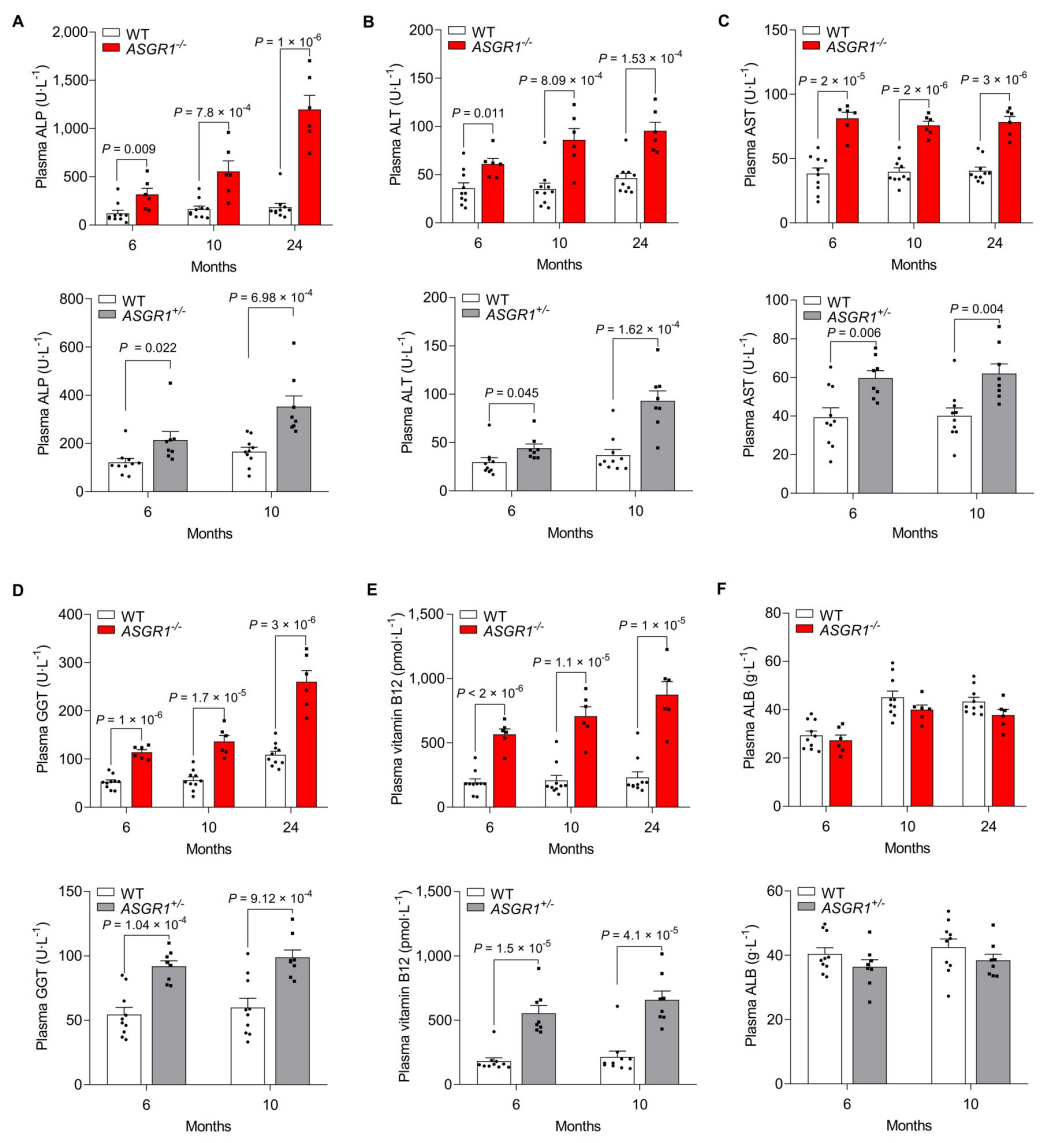
ASGR1-deficient pigs show high plasma levels of liver injury indicators. **A-F.** Plasma levels of **(A)** ALP, **(B)** ALT, **(C)** AST, **(D)** GGT, **(E)** vitamin B12 and **(F)** ALB in *ASGR1*^*-/-*^ pigs and age-matched WT pigs, as well as in *ASGR1*^*+/-*^ pigs. Points indicate data from individual pigs, at the indicated time points (*n* = 6 for *ASGR1*^*-/-*^ pigs, *n* = 8 for *ASGR1*^*+/-*^ pigs, *n* = 10 for WT pigs, data represent two independent experiments combined). *P* values are shown for indicated comparisons by the Student’s *t*-test. Values are mean ± SEM. The underlying data for this figure can be found in **S1 Data**. ALB, albumin; ALP, alkaline phosphatase; ALT, alanine aminotransferase; AST, aminotransferase; GGT, gamma glutamyl transpeptidase; WT, wild-type.

During GO-DisGeNET analysis of differentially-expressed genes (DEGs) in livers of 24-month-old *ASGR1*^*-/-*^ and their age-matched WT pigs, we observed a profile suggestive of liver injury in ASGR1-deficient pigs. To test whether ASGR1 deficiency affects liver function, we measured the levels of liver enzymes. Notably, the alkaline phosphatase (ALP) levels showed the most dramatic changes (**Fig 5A Upper panel**). At six months, we found an increase of 195.67 U per liter (161.71% higher) in *ASGR1*^*-/-*^ pigs than in WT controls. This difference increased to 390.79 U per liter (237.16% higher) at 10 months and increased further at 24 months. For *ASGR1*^*+/-*^ pigs and age-matched WT controls, we found an increase of 92.87 U per liter at six months and 186.64 U per liter at 10 months (**Fig 5A Lower panel**). These results indicate an age-dependent and gene dosage effect of ASGR1 deficiency on circulating ALP levels. Furthermore, we also observed mild increases in levels of alanine aminotransferase (ALT), aminotransferase (AST) gamma and glutamyl transpeptidase (GGT) in ASGR1-deficient pigs (**Fig 5B–5D**), but without a clear relationship to age or gene dosage. As in humans with loss-of-function *ASGR1* variants [15], *ASGR1*^*-/-*^ and *ASGR1*^*+/-*^ pigs showed elevated levels of plasma vitamin B12, a vitamin that is mainly stored in the liver (**Fig 5E**). Levels of albumin (ALB) were similar in ASGR1-deficient pigs and WT controls (**Fig 5F**).

ALP and GGT are glycoproteins with fractions of desialylated forms in the circulation [26]; they thus are putative endocytosis and degradation targets of ASGR1 based on the receptor’s known function [27]. To test whether ASGR1 deficiency causes accumulation of circulating desialylated glycoproteins including ALP and GGT, we measured serum levels of asialoglycoprotein and sialoglycoprotein using pig ELISA kits. ASGR1-deficient pigs showed higher asialoglycoprotein levels than WT controls (**S8A and S8B Fig**). To further examine the expression of the serum glycoproteins terminating with sialic acid- and with galactose, we performed lectin blots (Maackia amurensis agglutinin (MAA), Sambucus nigra agglutinin (SNA) and Ricinus communis agglutinin 120 (RCA120)) that detect terminal sugars of glycoproteins. ASGR1-deficient pigs showed similar patterns but different band intensities on the MAA and RCA 120 blots compared to WT controls (**S8C Fig**), suggesting that ASGR1 deficiency causes marked differences in plasma levels of asialoglycoprotein and sialoglycoprotein. These results show that ASGR1 deficiency causes extensive changes in circulating glycoprotein levels including ALP and GGT, indicative of liver injury.

### Mild to moderate liver injury was observed in ASGR1-deficient pigs

**Fig 6:**
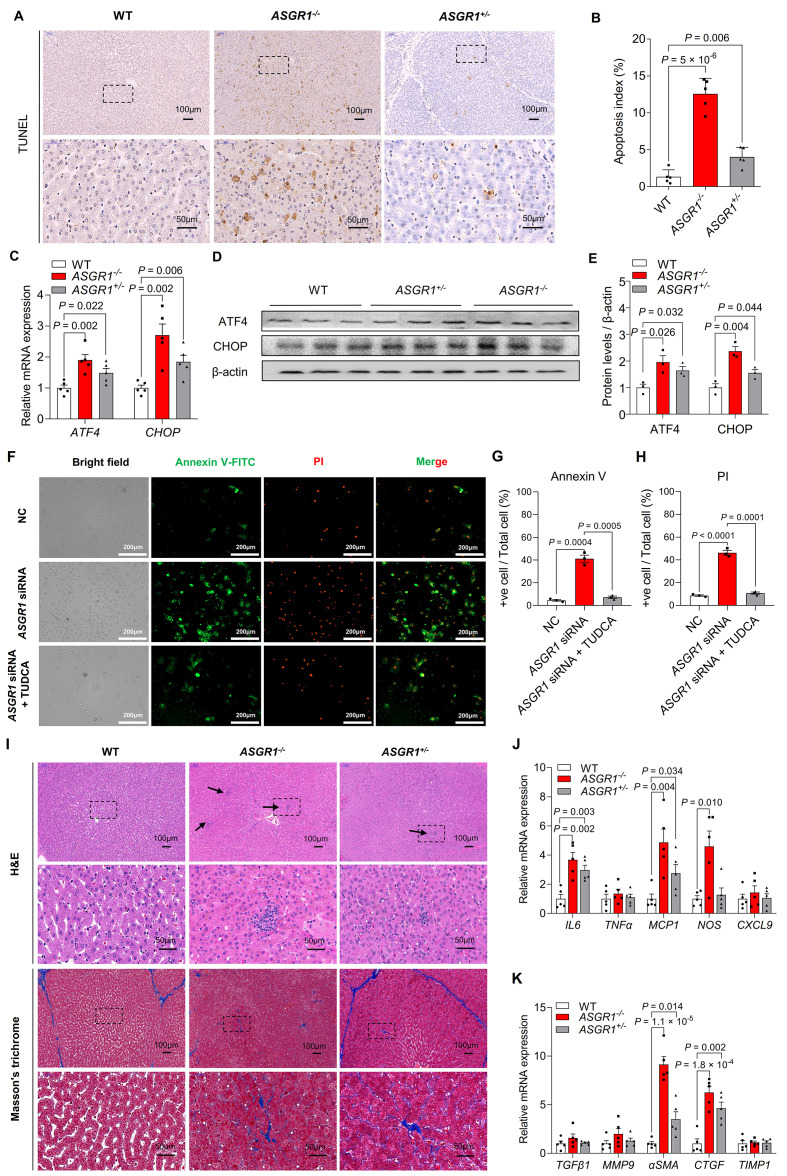
ASGR1-deficient pigs show liver injury. **A, B.** (**A**) Representative histological images of the TUNEL assay on liver tissue of 24-month-old WT and ASGR1-deficient pigs (Scale bars, 50 μm, 100 μm), (**B**) the apoptotic index quantification in livers of 24-month-old WT and *ASGR1*-deficient pigs (*n* = 5 per group, data represent two independent experiments combined). **C.** Relative mRNA expression of ER stress marker genes *ATF4* and *CHOP* in livers of pigs (*n* = 5 per group, data represent two independent experiments combined). **D, E.** Representative (**D**) Western blot analysis and (**E**) quantification of ATF4 and CHOP in the livers of pigs (*n* = 3 per group, data are representative of two independent experiments). **F.** Representative the fluorescence microscopy images of HepG2 cells treated with vehicle, 100 nM *ASGR1*-siRNA or 100 nM *ASGR1*-siRNA plus 400 μM TUDCA for 24 h, were stained with AV-FITC (green) and PI (red), and observed by confocal microscopy (×400). **G, H.** Quantification of the (**G**) AV-FITC positive cells and (**H**) PI positive nuclei compared with total HepG2 cells. Values are means ± SEM, (*n* = 3 per group, data are representative of three independent experiments). **I.** H&E and Masson’s trichrome staining of liver sections from 24-month-old pigs fed a normal diet. H&E staining showing inflammation (arrow) and Masson’s trichrome staining of the distribution of collagen in blue demonstrated positive fibrosis in the livers of ASGR1-deficient pigs (Scale bars, 50 μm, and 100 μm). **J**, **K.** Relative mRNA expression of (**J**) inflammation and (**K**) fibrosis-related markers in livers of ASGR1-deficient pigs and WT controls (*n* = 5 per group, data represent two independent experiments combined). *P* values are shown for indicated comparisons were analyzed using ordinary one-way ANOVA followed by Tukey’s test. Values are mean ± SEM. Points indicate data from individual pigs. The underlying data for this figure can be found in **S1 Data**. ANOVA, analysis of variance; ATF4, Activating Transcription Factor 4; AV-FITC, Apoptosis determined by Annexin V fluorescein isothiocyanate; CHOP, C/EBP homologous protein; ER, Endoplasmic reticulum; H&E, hematoxylin-eosin; PI, propidium iodide; TUDCA, tauroursodeoxycholic acid; TUNEL, terminal deoxynucleotidyl transferase dUTP nick end labeling; WT, wild-type.

To examine whether elevations in these clinical indicators (ALP, GGT, ALT and AST) upon ASGR1 deficiency reflect hepatic injury, we performed histological analysis and observed increased apoptosis in livers of ASGR1-deficient pigs (**Fig 6A and 6B**), accompanied by elevated mRNA and protein levels of the ER stress markers CHOP and ATF4 (**Fig 6C–6E**). These results are in accordance with the enriched terms of ‘apoptosis’ and ‘ER stress response’ by genes with expression changes between the livers of *ASGR1*^*-/-*^ pigs and WT controls (**Fig 4B**) Next, we investigated the effect of *ASGR1* on apoptosis in HepG2 cells and found higher apoptosis in *ASGR1* knockdown cells than in controls, as shown by increased double-positive staining of propidium iodide (PI) and Annexin V (**Fig 6F–6H**), suggesting that ASGR1 deficiency causes hepatocyte apoptosis. Since we observed the link between ER stress and apoptosis from transcriptomic analysis (**Fig 4B**) and experimental evidence (**Fig 6C–6E**), we predicted that ER stress might be a major cause of apoptosis. To test this hypothesis, *ASGR1* knockdown cells were further treated with the known ER stress inhibitor TUDCA [28]. As expected, inhibition of ER stress effectively attenuated *ASGR1* knockdown-induced apoptosis, suggesting that ER stress is a major contributor to ASGR1 deficiency-mediated hepatocyte apoptosis (**Fig 6F–6H**). Excessive hepatocyte apoptosis is associated with an increase in inflammation and liver fibrosis [29]. Consistent with this, we observed mild hepatic inflammation and fibrosis in ASGR1-deficient pigs (**Fig 6I**), accompanied by higher mRNA expression of inflammatory cytokines (*IL6*, *MCP1*, *NOS*) (**Fig 6J**) and fibrotic markers (*CTGF*, *α*-*SMA*) (**Fig 6K**). These findings suggest that ASGR1 may have a regulatory role in the development of liver disease.

## Discussion

Based on the finding that variant *ASGR1* in humans is associated with reduced levels of non-HDL-C and CAD [15], we generated an *ASGR1* knockout pig model using the CRISPR/Cas9 gene-editing approach. Deficiencies of ASGR1 significantly reduce plasma non-HDL-C and TG levels, and *ASGR1*^+/-^ pigs were strongly protected against HFHC diet-induced atherosclerosis. To the best of our knowledge, this is the first animal model that successfully recapitulates characteristic features of humans with *ASGR1* variants, confirming a causative role for ASGR1 deficiency in the protection of CVD. This work strongly suggests that reduction of ASGR1 could reduce cardiovascular disease in the general human population.

**Fig 7:**
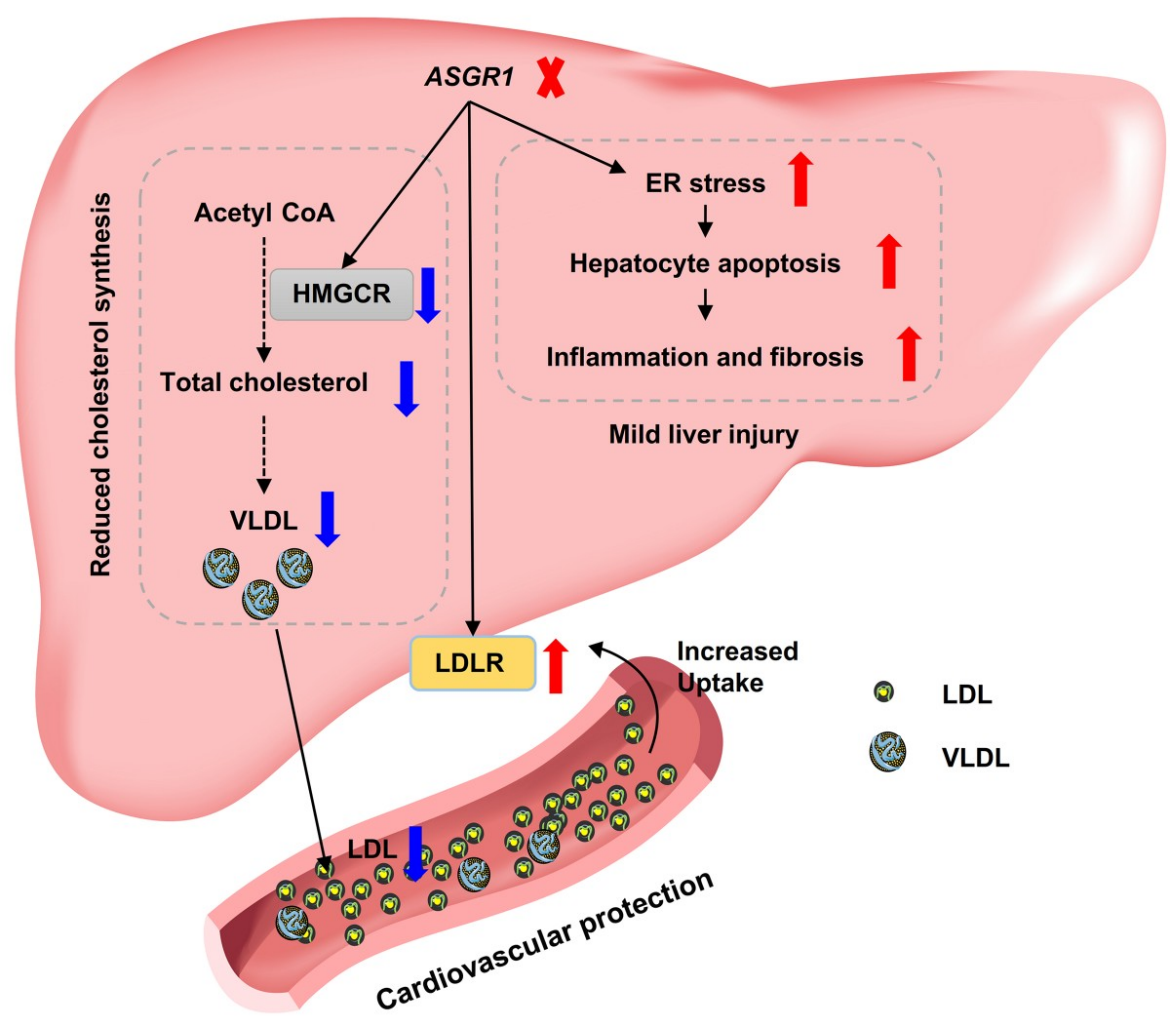
Graphical summary of the underlying mechanisms of ASGR1 deficiency-mediated atheroprotective effects and liver injury. **Left**, ASGR1 deficiency prevents atherosclerosis by downregulation of HMGCR-catalyzed hepatic cholesterol synthesis and its subsequent VLDL secretion, as well as by upregulation of LDLR-mediated hepatic LDL uptake. **Right,** the occurrence of liver injury shown by elevated hepatic enzymes, extensive apoptosis, mild inflammation and fibrosis due to ER stress-induced apoptosis upon ASGR1 deficiency.

We provide a mechanistic basis underlying this atheroprotective role using unbiased approaches and hypothesis-driven metabolic evidence. Both datasets pinpoint HMGCR, one of the clinical targets for hypercholesterolemia, as a critical regulator of this protection in ASGR1 deficiency. *In vivo* and *in vitro* results unequivocally support that ASGR1 deficiency reduced hepatic cholesterol synthesis through downregulation of HMGCR. Furthermore, ASGR1 deficiency increased hepatic LDLR protein levels and thus promoted LDL-C clearance (**Fig 7 left**). In regard to the elevated LDLR upon ASGR1 deficiency, we exclude a possible regulatory mechanism mediated by PCSK9, the well-established clinical regulator of LDLR [30,31], because there are comparable plasma levels of PCSK9 in ASGR1-deficient pigs and WT controls (**S9 Fig**). In line with our results, a recent finding shows that ASGR1 regulates LDLR levels and functions via post-translational regulation independently of PCSK9 in HepG2 cells [32]. They further demonstrated that ASGR1 physically binds and subsequently degrades LDLR. LDLR is an endocytic target for ASGR1. Whether this also occurs *in vivo* such as in humans and pigs warrants further investigation.

In humans, ASGR1 del12 variant has a larger effect on the risk of coronary artery disease than is predicted by its effect on levels of non-HDL cholesterol [15]. Consistent with this, we found that the degree of lipid lowering [LDL-C (36.27%), TC (18.58%), VLDL-C (21.98%), non-HDL-C (23.64%), TG (23.39%) and ApoB (14.64%)] (**Fig 2G**) appears disproportionally small to the degree of plaque reduction (74.18%) (**Fig 3I**) in pigs, indicating that besides lipid-lowering effects, there are other factors that play important roles in the plaque reduction by ASGR1 deficiency. ASGR1 is also expressed at low levels in macrophages and endothelial cells [21,33,34]. These cells also play pivotal roles in the development of atherosclerosis [35]. One recent study reported that ASGR1 affected the plaque formation and atherosclerosis by intimal macrophages [36]. In line with this, ASGR1-deficient pigs have lower numbers of macrophages in plaques compared to WT controls (**S3A–S3C Fig**) (1.15 ± 0.18% vs. 0.27 ± 0.07%, *P* = 0.011, *t*-test). Together, there data indicates that ASGR1 might also influence macrophage behaviors in plaque. These interesting points merit further investigation.

One important finding of our study is the occurrence of mild to moderate liver injury in ASGR1-deficient pigs. Several issues pertaining to this injury are worthy of discussion. First, higher levels of ALP and GGT, as well as a higher level of ALT (*P* = 0.065) were also found in humans with *ASGR1* variants [14]. However, the authors concluded that this possibly did not reflect the presence of liver disease because there was no association between *ASGR1* variants and serum levels of other indicators of liver function. Nonetheless, direct liver examination was not performed in these humans. Here, we confirmed mild liver injury in ASGR1-deficient animals, by histological evaluation of liver sections in pigs. More importantly, in line with our findings, Asgr1-deficient mice showed more severe liver injury and elevated hepatocyte death upon *Streptococcus pneumoniae* infection than did WT controls [37], supporting genetic susceptibility to liver injury in ASGR1 deficiency. Furthermore, reanalysis of data from our recent work [38] showed that, from a phylogenetic point of view, the liver exhibited a relatively lower evolutionary divergence in transcriptional output and resilience (with a shorter total branch length of expression-based trees [0.77]) than lung (0.88), adipose tissue (0.86), kidney (0.84) and spleen (0.79), and was comparable to heart (0.77) and higher than skeletal muscle [0.67]) among nine mammalian species (**S10A and S10B Fig**). Of the single-copy orthologous genes shared by nine mammalian species, *ASGR1* and genes spatially close to *ASGR1* exhibited relatively lower ΔAIC values and thus are relatively lower species-specific expression differences in the liver (variation in expression across species not due to evolutionary history, or expression changes after correcting the evolutionary nucleotide divergence between mammals) (**S11A and S11B Fig**). These results support the notion of an evolutionarily conserved regulatory mechanism of *ASGR1* in mammals. In humans, one genome-wide association study (GWAS) showed that single nucleotide polymorphism (SNP) (rs314253) within *ASGR1* was positively associated with ALP levels (*P* = 8.4×10^−12^), linking ASGR1 and the marker of liver injury [15]. Direct clinical evaluation of the presence and nature of liver injury is needed in humans.

The mechanisms underlying hepatic injury in ASGR1 deficiency may be partially attributable to ER stress-induced apoptosis and/or defective clearance ability of the liver (**Fig 7 right**). Extensive hepatic apoptosis is one characteristic feature of ASGR1 deficiency. We showed that ASGR1 knockdown causes hepatocyte apoptosis. ER stress, a well-characterized cause of apoptosis, contributes to this [39] (**Fig 4B**). Although ASGR1 is well-known as a cell surface receptor for endocytic degradation of circulating ligands, approximately 50% of the receptor was found to be in the ER [14] and its intracellular function has never been explored previously. By extension from the finding that ER-located LDLR was shown to eliminate prosecretory glycoprotein apoB [40], a similar clearance mechanism may also exist for ER-localized ASGR1. ASGR1 may be involved in the clearance of presecretory or misfolded glycoproteins in the ER, an intracellular rather than cell surface function of the receptor. These interesting and potentially important aspects of ASGR1 merit further investigation. Furthermore, a role for hepatocyte ASGPR in the clearance of apoptotic cells was shown by the ability of receptor-specific antibody and known ligands to block the uptake of apoptotic bodies [41]. Therefore, defective clearance of apoptotic cells by ASGR1-deficient hepatocytes might also contribute to the high number of apoptotic hepatocytes in ASGR1-deficent liver. In the long term, accumulation of apoptotic cells in the liver could lead to acute or chronic liver injury, with increased levels of ALP, GGT, ALT and AST in the circulation. Additionally, hepatic ASGPR was shown in rats to efficiently eliminate circulating desialylated glycoproteins, including ALP [42] and GGT [43], due to the cell surface receptor function of ASGPR. Thus, clearance of ALP and GGT would be impaired in the absence of ASGR1 in pigs and humans, which could be another reason for the observed elevated levels of ALP and GGT. Together, in ASGR1 deficiency, the observed elevations of circulating indicators of liver injury are likely due to both increased release from the liver and decreased hepatic clearance.

In conclusion, our results show that ASGR1 deficiency in pigs prevents or reduces CVD, supporting ASGR1 as a potential clinical target for the prevention and treatment of CVD in humans. Pharmaceutical ASGR1 inhibitors are currently under active development and testing by commercial companies. However, the unexpected liver injuries in ASGR1-deficient pigs under basal conditions could be accentuated in response to stresses such as alcohol abuse or external toxin insult. Of note, AMG 529, a human monoclonal antibody against ASGR1 that is in Phase 1 clinical trial for CVD treatment, is associated with dose-dependent elevations in ALP [44]. Careful elevation of the liver would seem prudent in ASGR1 deficiency or targeting. Altogether, our findings have important clinical implications both for cardiovascular and liver diseases. ASGR1-deficient pigs could be a valuable animal model to explore the underlying mechanisms of CVD as well as to test the genetic susceptibility and therapeutics for liver diseases.

## Materials and methods

### Ethics statement

All animal studies were approved by the Institutional Animal Care and Use Committee of the Northwest A & F University (approval number: NWAFU-314021167). All applicable institutional and/or national guidelines for the care and use of animals were followed, and we have complied with all relevant ethical regulations for animal experimentation.

### Animal study design

Bama miniature pigs were bred and raised at the Laboratory Animal Center of Chengdu Clonorgan Biotechnology Co. LTD. Pigs had free access to food and water unless otherwise specified. To accelerate the process of atherosclerosis, pigs were fed a HFHC diet (containing 20% saturated fat and 2% cholesterol, purchased from Chengdu Hualanxu Biotechnology Co. LTD, Chengdu, China) for six months.

We performed 4 studies in WT and ASGR1-deficient pigs (*ASGR1*^*-/-*^ and *ASGR1*^*+/-*^). Comparison of plasma lipoprotein profiles under normal diet (Study I), comparison of plasma lipoprotein profiles under HFHC diet (Study II), hepatic VLDL secretion assay after Poloxamer-407 injection (Study III), and lipoprotein clearance assay (Study IV).

To compare plasma lipoprotein profiles of WT and ASGR1-deficient pigs under normal diet, 10 WT and 6 age-matched *ASGR1*^*-/-*^ pigs were used at indicated time points (6, 10, 17 and 24 months), and a new cohort of 10 WT and 8 *ASGR1*^*+/-*^ pigs were used at indicated time points (4, 6, 10 and 17 months).

To compare plasma lipoprotein profiles of WT and *ASGR1*^*+/-*^ pigs under HFHC diet, six-month-old *ASGR1*^*+/-*^ pigs (*n* = 3) and age-matched WT controls (*n* = 3) were fed a HFHC diet containing 20% saturated fat and 2% cholesterol for six months. The schematic diagram of pig treatment was shown in **Fig 3A**.

Investigation of hepatic VLDL secretion in WT and *ASGR1*^*+/-*^ pigs. The hepatic VLDL secretion rate was measured in overnight fasted pigs (*n* = 8 in each group) by intraperitoneal injection of 1 g·kg^-1^ body weight Poloxamer-407 (P2164030, Sigma, MO, USA) as described in **S1 Text**. The blood samples were collected at 1 h, 2 h, 3 h and 4 h after administration of Poloxamer-407, and then plasma TC and TG levels were measured as described in **S1 Text**.

The lipoprotein clearance assay in WT and *ASGR1*^*+/-*^ pigs was performed as described in **S1 Text**. Each pig was intravenously injected with 0.35 mL·kg^-1^ lipoprotein fraction isolated from fresh plasma of WT and *ASGR1*^*+/-*^ pigs within 48 h of isolation. After injection, blood samples were collected at different times (0 min, 5 min, 1 h, 4 h, 12 h, 24 h, and 48 h). The clearance of ApoB-containing LDL particles was calculated based on plasma levels of ApoB measured by an ELISA kit (ml002356-1, Enzyme-linked Biotechnology, Shanghai, China) (*n* = 6 in each group).

## Methods

Detailed material and methods for Generation of ASGR1-deficient miniature pigs by CRISPR/Cas9, Plasma chemistry, Histological analysis, Lipoprotein clearance, Hepatic VLDL secretion assay, Total cholesterol content of the liver, Total triglyceride content of the liver, Cell culture and treatments, RNA isolation and real-time quantitative PCR, Transcriptome analysis, Western blotting, Lectin blot assay, Comparative transcriptomic of seven homologous tissues/organs for nine mammals and Statistical analysis are described in the **S1 Text**.

### Statistical analysis

Data are shown as the mean ± SEM. Statistical analysis were performed using GraphPad Prism 8 software. For analyses of intergroup differences between two groups, data were assessed using the Student’s *t*-test. For analysis of multiple comparisons, data were analyzed using ordinary one-way ANOVA followed by Tukey’s test.

## Supporting information

S1 Text Supplementary methods. (DOCX)

S1 Fig Genotype identification of ASGR1 knockout pig. Sequencing analysis revealed that three piglets (281,330,332) carried a 20 bp deletion (-20 bp / -20 bp), corresponding to donor A1 cells. Other three piglets (331,333,334) carried a 137bp deletion and 1 bp insertion (-137 bp /+1 bp), corresponding to donor A2 cells.
(TIF)

S2 Fig Representative images of Sudan IV-stained aortas of ASGR1-deficient pigs and WT controls. **A.** Representative images of Sudan IV-stained aortas of 24-month-old *ASGR1*^*-/-*^ founder pigs and age-matched WT controls fed a normal diet. **B.** Representative images of Sudan IV-stained aortic arch and thoracic aorta of *ASGR1*^*+/-*^ pigs and WT controls fed an HFHC diet. The area indicated by the black box is the area of the tissue used for histological assessment. HFHC, high-fat and high-cholesterol; WT, wild-type.
(TIF)

S3 Fig *ASGR1^+/-^* pigs reduced the numbers of macrophages in plaques under HFHC diet. Six-month-old *ASGR1*^*+/-*^ and age-matched WT pigs were fed an HFHC diet for six months. **A.** Immunofluorescence staining of CD68 in plaque from aortic arch of *ASGR1*^*+/-*^ and WT pigs. Scale bars, 100 μm. **B, C. (B)** Immunohistochemistry staining and **(C)** quantification of CD68 of plaque from aortic arch in *ASGR1*^*+/-*^ and WT pigs (*n* = 3 per group). Scale bars, 50 μm, 100 μm. Statistical analysis was performed using the Student’s *t*-test (*P* values are shown). Error bars show mean ± SEM. Points indicate data from individual pigs. The underlying data for this figure can be found in **S1 Data**. CD68, Cluster of Differentiation 68; HFHC, high-fat and high-cholesterol; WT, wild-type.
(TIF)

S4 Fig Metabolic profile of ASGR1-deficient pigs and WT controls. **A-C.** Six-month-old ASGR1-deficient pigs and age-matched WT controls fed a standard diet. **(A)** Body weights at 6,8,10 and 12 months. **(B)** Average food intake from 6 months to 12 months. **(C)** Body fat content at 24 months. **D, E.** Fecal content of **(D)** TC and **(E)** TG at 12months (*n* = 5 per group, data represent two independent experiments combined). **F.** Western blot of LIMA, NPC1L1 and β-actin (internal control) in the small intestine of 24-month-old ASGR1-deficient pigs and age-matched WT controls under normal diet. Statistical analysis was performed using the Student’s *t*-test (*P* values are shown). Error bars show mean ± SEM. Points indicate data from individual pigs. The underlying data for this figure can be found in **S1 Data**. LIMA1, LIM domain and actin-binding 1; NPC1L1, Niemann-Pick C1-Like 1; TC, total cholesterol; TG, triglycerides; WT, wild-type.
(TIF)

S5 Fig The mRNA expression profile of *ASGR1^-/-^* pigs and WT controls. **A, B.** Hepatic transcriptome profiling of three *ASGR*^*-/-*^ pigs and two WT controls. **(A)** Two-way t-SNE plot and **(B)** average linkage hierarchical clustering plot with Pearson’s correlation matrix based on the expression levels of mRNA. WT, wild-type.
(TIF)

S6 Fig Immunofluorescence of LDLR in livers from *ASGR1^-/-^* pigs and WT pigs. Representative immunofluorescence images of ASGR1 (green) and LDLR (red) in livers of *ASGR1*^*-/-*^ and WT pigs. **A-C.** The three different fields of the three biological replicates from two independent experiments. Scale bars, 100 μm. **D**. The quantification of LDLR fluorescence intensities in livers of *ASGR1*^*-/-*^ and WT pigs normalized by fluorescence values of three WT pigs (n = 3 per group, data are representative of two independent experiments). Statistical analysis was performed using the Student’s *t*-test (P values are shown). Error bars show mean ± SEM. Points indicate data from individual pigs. The underlying data for this figure can be found in **S1 Data**. ASGR1, asialoglycoprotein receptor 1; LDLR, low-density lipoprotein receptor; WT, wild-type.
(TIF)

S7 Fig Representative confocal immunofluorescence microscopy images of Dil-LDL uptake in HepG2 cells transfected with si-control or si-*ASGR1*. **A-C**. The images from the three independent experiments. Confocal microscopic images represent the fluorescence intensity of Dil-LDL (red) and DAPI (blue). Scale bars, 100 μm. DAPI, 4′, 6-diamidino-2-phenylindole.
(TIF)

S8 Fig The plasma glycoprotein levels of ASGR1-deficient pigs and WT controls. **A, B.** Serum levels of **(A)** asialoglycoprotein and **(B)** sialoglycoprotein were measured by ELISA in 12-month-old pigs fed a chow diet (*n* = 5 per group, data represent two independent experiments combined). **C.** Lectin blots of plasma proteins. Blood was collected from 12-month-old WT or ASGR1-deficient pigs. Lectin blots of plasma proteins using MAA, SNA and RCA120 conjugated with biotin. Biotin conjugates were respectively detected by HRP-labeled anti-biotin antibodies. Statistical analysis was performed using the Student’s *t*-test (*P* values are shown). Error bars show mean ± SEM. Points indicate data from individual pigs. The underlying data for this figure can be found in **S1 Data**. MAA, Maackia amurensis agglutinin; RCA120, Ricinus communis agglutinin 120; SNA, Sambucus nigra agglutinin; SNP, single nucleotide polymorphism; WT, wild-type.
(TIF)

S9 Fig Plasma levels of PCSK9 in ASGR1-deficient pigs and WT controls. Plasma levels of PCSK9 was measured by ELISA in 12-month-old pigs fed a standard diet (*n* = 5 per group, data represent two independent experiments combined). Statistical analysis was performed using the Student’s *t*-test (*P* values are shown). Error bars show mean ± SEM. Points indicate data from individual pigs. The underlying data for this figure can be found in **S1 Data**. PCSK9, proprotein convertase subtilisin/kexin type 9; WT, wild-type.
(TIF)

S10 Fig Gene expression phylogenies for seven tissues/organs across nine mammals. **A.** Gene expression phylogenies for seven tissues/organs across nine mammalian species. A neighbor-joining expression tree was constructed based on 1-Spearman correlation distances of 3,602 single-copy orthologous genes identified in all of the nine mammals. **B.** Box plot depicting the total branch lengths of neighbor-joining expression trees (as shown in **S10A Fig**) constructed based on pairwise (1-*r*) (*r*: Spearman’s correlation coefficient) distances across nine mammals for each tissue/organ. The lines inside denote the median value.
(TIF)

S11 Fig Species-specific expression shifts in liver across nine mammalian models. **A.** Histograms representing the distribution of ΔAIC values. The black vertical dashed lines indicate the ΔAIC value of *ASGR1*. **B.** Box plot depicting the distribution of ΔAIC values of genes that are spatially close to (blue) and distant from (red) *ASGR1* in the liver across nine mammals. To eliminate bias effects when comparing two populations with large magnitude differences, we randomly selected non-*ASGR1* single copy orthologous genes that are spatially close to *ASGR1* and calculated the median ΔAIC value. We repeated this process 10,000 times. The Wilcoxon rank sum test was then performed to compare ΔAIC values between *ASGR1* and its spatially close genes, and randomly generated median values.
(TIF)

S12 Fig Original blots. (TIF)

S1 Table Identification of ASGR1 knockout colonies. (DOCX)

S2 Table Efficiency of SCNT in generating ASGR1-deficient pigs. (DOCX)

S3 Table The number of ASGR1-deficient pigs in each generation. (DOCX)

S4 Table Results of off-target analysis. (DOCX)

S5 Table Primers used for off-target analysis. (DOCX)

S6 Table Primers used for gene expression analysis. (DOCX)

S7 Table Antibody information. (DOCX)

S1 Data Contains underlying data for Figs 2A–2H; 3B–3F, 3H–3I; 4C, 4E–4M, 4O; 5A–5F; 6B–6C, 6E, 6G–6H, 6J–6K; S3C; S4A–S4E; S6B; S8A–S8B; S9. (XLSX)

S2 Data Summary of the RNA-seq data of liver. (XLSX)

S3 Data Summary of downloaded RNA-seq data used in this study. (XLSX)
