# Supplementary material for: Deficiency of ASGR1 in pigs recapitulates reduced risk factor for cardiovascular disease in humans
Source: PLoS Genet. 2021 Nov 11;17(11):e1009891. doi: 10.1371/journal.pgen.1009891 (PMC8584755; doi:10.1371/journal.pgen.1009891)
Supplement: S1 Text — (DOCX) [file pgen.1009891.s001.docx]

# Supplementary methods

# Generation of ASGR1-deficient miniature pigs by CRISPR/Cas9

*CRISPR/Cas9 vector construction*

We designed two sgRNAs targeting exon 6 of the porcine *ASGR1* gene (Gene ID: 100625649) using online tools (http:crispr.mit.edu/). SgRNA sequences are 5’-caccgagcagtttgtgtccgacctg-3’ (sgRNA-1) and 5’-caccgcttcacgtggagcagcaagc-3’ (sgRNA-2). The complementary oligo DNA of sgRNAs (GENEWIZ, Beijing, China) was annealed to double-stranded DNA and cloned downstream of the human U6 promoter in the *BbsI* restriction site of plasmid pX330-U6-Chimeric_BB-CBh-hSpCas9 (# 42230, Addgene) to generate the Cas9-sgRNA plasmids targeting ASGR1.

*Isolation and culture of ﬁbroblasts*

Fibroblasts were isolated from ear biopsies of a five-month-old male Bama miniature pig and maintained in culture medium [Dulbecco’s modiﬁed Eagle’s medium (DMEM) supplemented with 10% fetal bovine serum (FBS), MEM nonessential amino acids (Life Technologies)], 1 mM sodium pyruvate, 60 µg·mL^-1^ penicillin, and 100 µg·mL^-1^ streptomycin), at 37 °C in a humidiﬁed incubator containing 5% CO_2_.

*Transfection and selection*

To determine the cleavage efficiency of the Cas9-sgRNAs, the constructed Cas9-sgRNA plasmids were transfected into pig ear fibroblasts. Cleavage efficiency was tested by PCR amplification and the resultant fragments were subjected to TA cloning for Sanger sequencing. The InDels induced by sgRNA1 reached 22% (9/40), and no effect was observed for sgRNA2. Therefore, sgRNA1 was used in subsequent experiments. To establish ASGR1-knockout cell lines, *ASGR1* Cas9-sgRNA1 targeting plasmids were used for transfection and selection. Specifically, 4 μg of targeting plasmid was cotransfected with 1 μg of the EGFP-expression plasmid (Clontech Laboratories, Mountain View, CA, USA) into 1 × 10^6^ early passages of suspended Bama miniature pig ear fibroblasts by Procedure T-016 using the Amaxa Nucleo-factor II transfection Instrument (Amaxa, Cologne, Germany) according to the manufacturer's instructions. The EGFP-expression plasmid was used to trace and enrich the transfected cells. The post-transfection cells were incubated in DMEM supplemented with 20% FBS (Gibco, NY, USA) at 37°C in an incubator with 5% CO_2_. After 48 h of recovery, cells were digested with trypsin (Sigma, MO, USA) and resuspended in PBS (Gibco, Grand Island, NY, USA). Cells expressing green fluorescence were enriched by fluorescence-activated cell sorting (FACS) using an Aria II Cell Sorter (BD Biosciences, CA, USA). Five thousand of these cells were used for detecting mutation efficiency, and the remaining cells were seeded in 100 mm plates (100 cells/plate). Cells were cultured for 12 d to obtain single colonies. The single colonies were passed into 48-well plates respectively and cultured until confluence. 20% of cells were lysed for genotyping (the primers are listed in **Fig 1A**). PCR conditions were as follows: 95°C for 5 min, followed by 35 cycles of 95°C for 5 s, 60°C for 30 s, 72°C for 60 s, with a final 72°C for 10 min. The PCR products were subcloned into a pMD18-T vector (Takara, Tokyo, Japan), and 18 individual colonies were picked and sequenced. Colonies with ideal knockout types were selected and used for somatic cell nuclear transfer (SCNT).

*SCNT and animal breeding*

To obtain cloned pig embryos, we collected porcine oocytes from the ovaries of a local abattoir. Cumulus-oocyte complexes (COCs) were cultured in maturation medium for 42-44 h *in vitro*. Then the nucleus and polar body were removed from the mature oocytes with an enucleation pipette, and the *ASGR1* knockout cells were injected into the perivitelline space of oocytes. We selected sows during estrus as embryo transfer recipients and transplanted the reconstructed embryos into the oviduct of the recipient sows as previously described [1]. Pregnancy was examined by ultrasound 30 days after transplantation and monitored until the perinatal period. All cloned piglets were delivered by natural birth. Male and female F1 generation pigs (*ASGR1^+/-^*) were crossed to obtain F2 generation pigs.

*Genotyping of cloned pigs*

Genomic DNA was extracted from punched ear tissue of newborn gene-edited pigs using the DNeasy Blood and Tissue Kit (Qiagen, CA, USA). Genotyping was detected by PCR amplification as described above. The PCR products were subsequently cloned into pMD18-T vectors (Takara, Kyoto, Japan) for Sanger sequencing.

*Off-target analysis*

To detect whether off-target mutagenesis occurred in ASGR1-deficient piglets, potential off-target sites of sgRNA1 were predicted by screening the pig genome using an online tool (<http://crispr.mit.edu/>) based on sequence homology to the *ASGR1* sgRNA sequence, allowing for alignments with up to 4 mismatches. To verify whether a total of ten predicted off-target sites (**S4Table**) cleavage events occurred in the obtained ASGR1-deficient piglets, PCR amplification (primers listed in **S5 Table**) and Sanger sequencing of these sites were performed.

# Plasma chemistry

Plasma was collected from the jugular vein of the overnight-fasted pigs (16 h) and then separated to analyze levels of various substances that were measured according to standard procedures using the biochemical autoanalyzer (Hitachi 7180 automated analyzer, Tokyo, Japan) in the Yang-ling Demonstration District Hospital of Shaanxi Province, including TC, HDL-C, LDL-C, VLDL-C, TG, ApoA1, ApoB, ALB, ALT, AST, GGT, ALP and vitamin B12. Plasma lathosterol levels were determined by gas-liquid chromatography as previously described [2]. Plasma levels of asialoglycoprotein (ASGP) (ml308144, Enzyme-linked Biotechnology, Shanghai, China) and sialoglycoprotein (SGP) (ml612197, Enzyme-linked Biotechnology, Shanghai, China) were detected using ELISA kits according to the manufacturer’s protocol.

# Histological analysis

A*rtery histology analysis*

Aortic atherosclerotic lesions of pigs fed a HFHC diet were assessed as previously described [3]. In brief, the arteries were carefully dissected, excised, and fixed in 10% formalin at 4°C overnight before processing and embedding in paraffin. All specimens were cut into 5 µm-thick sections. For evaluation of gross atherosclerotic lesions, the sections of the aorta were dissected and en face stained with Sudan IV. For morphology and delineation of elastin layers, the sections were stained with hematoxylin and eosin (H&E) (G1005, Servicebio, Hubei, China) or elastic van Gibson (EVG) (G1042, Servicebio, Hubei, China), respectively. For microscopic evaluation of the cellular components of the lesions, sections of the aorta were Immunofluorescence and immunohistochemically stained with anti-CD68 (GB11067, Servicebio, Hubei, China) and Immunofluorescence and anti-ASGR1 (sc-393849, Santa Cruz Biotechnology, Santa Cruz, CA, USA) incubated for 1 h with secondary antibodies (1:1,000) at room temperature. The artery lesions lumen area was quantified under a light microscope [(lesion area/total lumen area) x 100%].

*Liver histology analysis*

Tissue fragments were fixed in 4% paraformaldehyde tissue overnight before processing and embedding in paraffin. All specimens were cut into 5 µm-thick sections and collected. The sections were stained for H&E, Masson's trichrome (G1006, Servicebio, Hubei, China), and terminal deoxynucleotidyl transferase dUTP nick end labeling (TUNEL) (11684817910; Roche, Mannheim, Germany) as previously described [4]. Quantification of TUNEL-positive (TUNEL^+^) cells in the liver section was performed with ImageJ software. In each section, the numbers of (TUNEL^+^) cells in six randomly selected high-power fields (200×) were counted. Sections of the liver were immunofluorescence and immunohistochemically stained with anti-LDLR (ab30532, Abcam, Cambridge, UK) and Immunofluorescence and anti-ASGR1 (sc-393849, Santa Cruz Biotechnology, Santa Cruz, CA, USA) incubated for 1 h with secondary antibodies (1:1,000) at room temperature.

# Lipoprotein clearance

The lipoprotein clearance assay in WT and *ASGR1^+/-^* pigs was performed as described [5]. Briefly, porcine LDLs and HDLs were isolated from fresh plasma (*n* = 10 donors) by ultracentrifugation at 59,000 rpm for 18 h in a 60Ti rotor at density 1.02 to 1.06 g·mL^-1^, and then the lipoprotein fraction was dialyzed against 0.9 % saline at 4ºC for 16 h [6]. Each pig was intravenously injected with 0.35 mL·kg^-1^ lipoprotein fraction isolated from fresh plasma of healthy WT pigs within 48 h of isolation (*n* =10). After injection, blood samples were collected at different times (0 min, 5 min, 1 h, 4 h, 12 h, 24 h, and 48 h). The clearance of ApoB-containing LDL particles was calculated based on plasma levels of ApoB measured by an ELISA kit (ml002356-1, Enzyme-linked Biotechnology, Shanghai, China).

# Hepatic VLDL secretion assay

The hepatic VLDL secretion rate in overnight fasted pigs was measured by intraperitoneal injection of 1 g·kg^-1^ body weight Poloxamer-407 (P2164030, Sigma, MO, USA) as previously described [7]. The blood samples were collected at 1 h, 2 h, 3 h and 4 h after administration of Poloxamer-407, and then plasma cholesterol and TG levels were measured as described above.

# Total cholesterol content of the liver

The TC content of the liver was measured as previously described [8]. The liver sample was homogenized in 0.9% NaCl and then extracted for 1 h with shaking in a mixture of chloroform-methanol (2:1) plus 4 mL of 0.9% NaCl. After centrifugation for 10 min at 2,000 g, the chloroform phase was removed and then evaporated under nitrogen. Lipids were resuspended in chloroform-methanol (2:1), and thin-layer chromatography (TLC) was performed (Partisil K5; Whatman International Ltd., Maidstone, England) using hexane-ether-acetic acid (80:20:3) as the mobile phase. Scrapings of the cholesterol spots were extracted with chloroform-methanol (2:1) and quantified using the Cholesterol Assay Kit (ab65359, Abcam, CA, USA).

# Total triglyceride content of the liver

Total triglyceride content of the liver was measured by using the assay kit (Jiancheng Biotechnology, Nanjing, China) according to the manufacturer’s instructions.

# Cell culture and treatments

HepG2 cells were cultured as previously described [9], and were treated with tauroursodeoxycholic acid (TUDCA, 400 μmol·L^-1^, an ER stress inhibitor) (T0266, Sigma, MO, USA) for 24 h in some experiments. The isolation and primary culture of porcine hepatocytes were performed as previously described [10]. Briefly, the liver was perfused with 1 L of Dulbecco’s phosphate-buffered saline (DPBS) without Ca^2+^ and Mg^2+^ supplemented with 10 mM HEPES. Then, the liver was cut into small pieces and digested with 0.025% Type II collagenase under gentle stirring for 15 min. The cell suspension was poured into a sieve and stirred with a glass rod while filtering. Large pieces of liver tissue were removed using a coarse sieve, followed by filtering with a 100-mesh filter. Hepatocytes were then isolated by a 5 min centrifugation at 100 g. After the cells were isolated, they were seeded directly according to the procedure described below.

*siRNA transfection*

siRNA transfections were performed according to the manufacturer’s instructions (G04002, GenePharma, Shanghai, China). Briefly, cells were cultured in DMEM containing 10% FBS and seeded in 6-well plates at a density of 3 × 10^6^ cells/well. The following day, cells were transfected with 100 nM *ASGR1* siRNA 5’-GCUGCUUGUGGUUGUCUGUTT-3’ or negative-control siRNA 5’-UUCUCCGAACGUGUCACGUTT-3’ mixed with transfection reagent and incubated at 37 °C for 72 h. Each siRNA transfection was performed in triplicate.

*Cholesterol de novo synthesis assay*

The cholesterol *de novo* synthesis assay in primary porcine hepatocytes and HepG2 cells was performed as previously described [11]. Briefly, the cells were incubated in fresh serum-free medium with 2 μCi of tritium-labelled acetic acid (NET003, Perkin Elmer, MA, USA) and 2.5 mM sodium acetate (S5636, Sigma, MO, USA) for 4 hours at 37°C. A fraction of cell lysate was used for protein determination. Then, the lipids were isolated from the cell lysate using the Folch method, to which was added 0.045 μCi of ^14^C radiolabeled cholesterol (NEC018050UC, Perkin Elmer, MA, USA). TLC was performed. The cholesterol fraction was isolated and scintillation counting was performed. The measurement of newly synthesized cholesterol was normalized for the amount of initial protein and for ^14^C counts (internal control for extraction efficiency).

*Dil-LDL uptake assay*

The LDL-C uptake was determined using fluorescently labeled Dil-LDL (L3482, Thermo Fisher Scientific, MA, USA) in HepG2 cells according to the manufacturer’s protocol. Briefly, HepG2 cells were cultured in serum-free medium for 24 h and then incubated in serum-free medium containing 5 mg·mL^-1^ Dil-LDL for 4 h in the dark. Then, cells were stained with DAPI for 20 min and washed 3 times with PBS. The images were obtained with a fluorescence microscope (Spinning Disk Confocal Microscope, Revolution WD, Andor, England). The Dil-LDL-stained cell membrane showed orange-red fluorescence, and the DAPI-stained cell nucleus showed blue fluorescence.

*Assessment of cellular apoptosis by Annexin V-FITC / PI dual staining*

Apoptosis of HepG2 cells transfected with *ASGR1* siRNA or negative-control siRNA was detected by using the Annexin V-FITC Detection Kit (C1062M, Beyotime Biotechnology, Shanghai, China) according to the manufacturer’s protocol. Briefly, the cells were washed and subsequently incubated for 15 min at room temperature in the dark in 100 μL of 1x binding buffer containing 5 μL of Annexin V-FITC and 10 μL of propidium iodide (PI). Confocal laser imaging was obtained (Confocal Microscope, Revolution WD, Andor, England).

# RNA isolation and real-time quantitative PCR

Total RNA was extracted using TRIzol reagent (9109, Takara, Kyoto, Japan) and then reverse-transcribed into cDNA using Prime Script™ RT reagent Kit (RR047A, Takara, Kyoto, Japan) according to the manufacturer's instructions. Real-time quantitative PCR was performed using the ABI 7900 HT real-time PCR (RT-PCR) amplification complementary DNA system with the specific primers listed in **S6** **Table**. Relative mRNA levels were quantified by calculating the comparative 2^-ΔΔCt^ method [12].

# Transcriptome analysis

*RNA-seq libraries*

Total RNA was extracted from the liver tissue of 5 samples (2-year-old male WT Bama pigs and three 2-year-old male *ASGR1*^-/-^ Bama pigs) using the RNeasy Mini Kit (Qiagen, CA, USA). We used oligo (dT) magnetic beads to isolate mRNA. The Agilent 2100 Bioanalyzer and ABI StepOnePlus RT-PCR System were used for quantification. The libraries were then sequenced on the BGISEQ-500 platform. Detailed information shown in **S2 Data**.

*Gene expression quantification*

In total, the RNA-seq data were aligned to corresponding reference genomes (Sscrofa11.1) by using STAR (V2.3.0, http://code.google.com/p/rna-star/) and applied a novel strategy for spliced alignments [13]. The gene expression level was then estimated as transcripts per million (TPM) using the high-speed transcript quantification tool [14] (V0.43.0, <https://pachterlab.github.io/kallisto/>).

*Identification of differentially expressed genes*

To identify DEGs between liver tissue of *ASGR1* knockout pigs and WT pigs, we used the edgeR package, which implemented novel statistical methods based on the negative binomial distribution as a model for count variability, including empirical Bayes methods, exact tests and generalized linear models. Genes with false discovery rate (FDR) < 0.05 and |fold change| > 2 were considered DEGs.

*Functional enrichment analysis of genes*

Gene datasets in pigs were first converted to the human Ensembl accession ID. Functional enrichment analysis of Gene Ontology (GO) terms and pathways was performed using A Gene Annotation & Analysis Resource (Metascape) [15]. Only GO-biological process (GO-BP) and DisGeNET with a *P* value less than 0.05 were considered significant and listed.

# Western blotting

Tissue samples were homogenized in lysis buffer (P0013C, Beyotime Biotechnology, Shanghai, China), and the protein concentration was determined by the Bradford method (5000001, Bio-Rad, CA, USA). Western blots were performed as described [16] with antibodies summarized in **S7 Table**.

# Lectin blot assay

Lectin blot analysis was performed as previously described [17]. One microliter of plasma was separated by 4-15% SDS polyacrylamide gel electrophoresis under reducing conditions and transferred to polyvinylidene difluoride membranes. Lectin blotting was performed using *Maackia amurensis* agglutinin, which is a lectin specific for NeuNAc (#2-3) Gal on N-linked carbohydrate conjunct with biotin (MAA, B-1315-2, Vector Laboratories, CA, USA), *Sambucus nigra* agglutinin, which is a lectin specific for NeuNAc (#2-6) Gal on N-linked carbohydrate conjugated with biotin (SNA, B-1305-2, Vector Laboratories, CA, USA), and *Ricinus communis* agglutinin 120, which is a lectin specific for galactose conjunct with biotin (RCA120, B-1085-5, Vector Laboratories, CA, USA). The biotin was detected by horseradish peroxidase-labeled anti-biotin antibody (A0303, Beyotime Biotechnology, Shanghai, China).

# Comparative transcriptomics of seven homologous tissues / organs for nine mammals

*Mapping and quantification*

To explore the evolutionary divergence of the transcriptome contributing to the specific tissue biology of mammals, we downloaded publicly available RNA-seq data of seven biologically important homologous organs/tissues (adipose, kidney, liver, lung, skeletal muscle, spleen) from pigs and eight mammals that represent major mammalian models (nonhuman primates [rhesus], rodents [mouse, rat, and guinea pig], lagomorphs [rabbit], carnivora [dog and cat], and artiodactyla [sheep]) [18] and performed a comparative transcriptomics analysis **(S3 Data)**.

Raw sequencing data of a total of 121 rRNA-depleted RNA-seq libraries were downloaded from the NCBI Gene Expression Omnibus (GEO) under accession numbers SRR11939363 to SRR11939370, SRR11939372 to SRR11939381, SRR11939383 for macaque, SRR11939355 to SRR11939359, SRR11939361 and SRR11939362 for mouse, SRR11939301 to SRR11939302, SRR11939304 to SRR11939308 for rat, SRR11939159, SRR11939170, SRR11939181, SRR11939192, SRR11939203, SRR11939214 and SRR11939225 for guinea pig, SRR11939331 to SRR11939346, SRR11939350 to SRR11939354 for rabbit, SRR11939407 to SRR11939414, SRR11939416 to SRR11939423, SRR11939434, SRR11939445, SRR11939457 and SRR11939468 for cat, SRR11939236, SRR11939237, SRR11939248, SRR11939259, SRR11939270, SRR11939281, SRR11939292, SRR11939303, SRR11939314, SRR11939325, SRR11939336, SRR11939347- SRR11939349, SRR11939360, SRR11939371, SRR11939382, SRR11939393, SRR11939404, SRR11939415 and SRR11939446 for dog, SRR11939309 to SRR11939313, SRR11939315 to SRR11939324, SRR11939326 to SRR11939330 for sheep.

Reviewer’s link:

https://dataview.ncbi.nlm.nih.gov/object/PRJNA637678?reviewer=aofh09l1omgdpqej6ds0ul0n.

These RNA-seq data were aligned to their respective reference genomes (monkey [Mmul_8.0.1], mouse [GRCm38], rat [Rnor_6.0], guinea pig [Cavpor3.0], rabbit [OryCun2.0], cat [Felis_catus_6.2], dog [CanFam3.1] and sheep [Oar_v3.1]) using STAR. The protein-coding genes were quantified by Kallisto **(see Transcriptome analysis Methods for details)**.

Identification of single-copy ortholog protein-coding genes

Single-copy orthologous genes were identified following a protocol similar to the pipeline recommended by Ensembl (<http://asia.ensembl.org/info/genome/compara/homology_method.html>). In brief, first, the longest protein-coding translation was extracted for each gene of each species, then an all-against-all BLAST of these translations was performed between both self and nonself-species; second, based on BLAST results, a sparse graph was built, and clusters were extracted using hclust_sg; third, large clusters with over 400 genes were split into smaller ones recursively until no cluster with 400 genes was retained; fourth, for each cluster, multiple alignments of protein-coding sequences were constructed and further back-translated to CDS alignments; finally, a phylogenetic tree was built, and single-copy orthologous gene families were identified.

*Construction of expression-based trees for homologous tissues/organs*

We constructed neighbor-joining expression trees for each tissue across nine mammals based on pairwise (1-r) (r: Spearman’s correlation coefficient) distances of single-copy orthologs and extracted the total branch length representing the evolutionary speed for each tissue.

*Characterizing patterns of selection on gene expression*

First, species trees with topology and branch lengths were retrieved from TimeTree. We characterized the pattern of expression shifts for each gene as previously described [19]. In brief, we fitted a single optimum OU (Ornstein-Uhlenbeck) model using the R package geiger and BM1 (single-rate Brownian)/BM2 (multi-rate Brownian) models using the ‘brownie.lite’ function in the R package phytools to the cross-species expression data for each tissue and the species tree. The expression shifts for each gene were characterized by the ΔAIC value (the AIC value of the BM1 or OU model minus that of the BM2 model, whichever is smaller).

# S **References**

1. Pan D, Zhang Y, Sun X, Zhang J, Li X, Li Y, et al. Cloned pigs derived from somatic cell nuclear transfer embryos cultured in vitro at low oxygen tension. Chinese Science Bulletin. 2006;51(7):839-44.

2. Gelzo M, Di Taranto MD, Sica C, Boscia A, Papagni F, Fortunato G, et al. Age-related changes of cholestanol and lathosterol plasma concentrations: an explorative study. Lipids Health Dis. 2019;18(1):235. doi: 10.1186/s12944-019-1176-3. PubMed PMID: 31888647; PubMed Central PMCID: PMCPMC6937658.

3. Fang B, Ren X, Wang Y, Li Z, Zhao L, Zhang M, et al. Apolipoprotein E deficiency accelerates atherosclerosis development in miniature pigs. Dis Model Mech. 2018;11(10). doi: 10.1242/dmm.036632. PubMed PMID: 30305304; PubMed Central PMCID: PMCPMC6215431.

4. Xia B, Cai GH, Yang H, Wang SP, Mitchell GA, Wu JW. Adipose tissue deficiency of hormone-sensitive lipase causes fatty liver in mice. PLoS Genet. 2017;13(12):e1007110. doi: 10.1371/journal.pgen.1007110. PubMed PMID: 29232702; PubMed Central PMCID: PMCPMC5741266.

5. Al-Mashhadi RH, Sorensen CB, Kragh PM, Christoffersen C, Mortensen MB, Tolbod LP, et al. Familial hypercholesterolemia and atherosclerosis in cloned minipigs created by DNA transposition of a human PCSK9 gain-of-function mutant. Sci Transl Med. 2013;5(166):166ra1. doi: 10.1126/scitranslmed.3004853. PubMed PMID: 23283366.

6. Tall AR, Atkinson D, Small DM, Mahley RW. Characterization of the lipoproteins of atherosclerotic swine. J Biol Chem. 1977;252(20):7288-93. PubMed PMID: 198408.

7. Yu H, Rimbert A, Palmer AE, Toyohara T, Xia Y, Xia F, et al. GPR146 Deficiency Protects against Hypercholesterolemia and Atherosclerosis. Cell. 2019;179(6):1276-88 e14. doi: 10.1016/j.cell.2019.10.034. PubMed PMID: 31778654; PubMed Central PMCID: PMCPMC6889877.

8. Li T, Matozel M, Boehme S, Kong B, Nilsson LM, Guo G, et al. Overexpression of cholesterol 7alpha-hydroxylase promotes hepatic bile acid synthesis and secretion and maintains cholesterol homeostasis. Hepatology. 2011;53(3):996-1006. doi: 10.1002/hep.24107. PubMed PMID: 21319191; PubMed Central PMCID: PMCPMC3079544.

9. Woudenberg J, Rembacz KP, Hoekstra M, Pellicoro A, van den Heuvel FA, Heegsma J, et al. Lipid rafts are essential for peroxisome biogenesis in HepG2 cells. Hepatology. 2010;52(2):623-33. doi: 10.1002/hep.23684. PubMed PMID: 20683960.

10. Zhou X, Tokiwa T, Kano J, Kodama M. Isolation and primary culture of adult pig hepatocytes. Methods in cell Science. 1998;19(4):277-84.

11. Lai Q, Giralt A, Le May C, Zhang L, Cariou B, Denechaud PD, et al. E2F1 inhibits circulating cholesterol clearance by regulating Pcsk9 expression in the liver. JCI Insight. 2017;2(10). doi: 10.1172/jci.insight.89729. PubMed PMID: 28515357; PubMed Central PMCID: PMCPMC5436545.

12. Livak KJ, Schmittgen TD. Analysis of relative gene expression data using real-time quantitative PCR and the 2(-Delta Delta C(T)) Method. Methods. 2001;25(4):402-8. doi: 10.1006/meth.2001.1262. PubMed PMID: 11846609.

13. Dobin A, Davis CA, Schlesinger F, Drenkow J, Zaleski C, Jha S, et al. STAR: ultrafast universal RNA-seq aligner. Bioinformatics. 2013;29(1):15-21. doi: 10.1093/bioinformatics/bts635. PubMed PMID: 23104886; PubMed Central PMCID: PMCPMC3530905.

14. Bray NL, Pimentel H, Melsted P, Pachter L. Near-optimal probabilistic RNA-seq quantification. Nat Biotechnol. 2016;34(5):525-7. doi: 10.1038/nbt.3519. PubMed PMID: 27043002.

15. Zhou Y, Zhou B, Pache L, Chang M, Khodabakhshi AH, Tanaseichuk O, et al. Metascape provides a biologist-oriented resource for the analysis of systems-level datasets. Nat Commun. 2019;10(1):1523. doi: 10.1038/s41467-019-09234-6. PubMed PMID: 30944313; PubMed Central PMCID: PMCPMC6447622.

16. Xia B, Shi XC, Xie BC, Zhu MQ, Chen Y, Chu XY, et al. Urolithin A exerts antiobesity effects through enhancing adipose tissue thermogenesis in mice. PLoS Biol. 2020;18(3):e3000688. doi: 10.1371/journal.pbio.3000688. PubMed PMID: 32218572; PubMed Central PMCID: PMCPMC7141696.

17. Tozawa R, Ishibashi S, Osuga J, Yamamoto K, Yagyu H, Ohashi K, et al. Asialoglycoprotein receptor deficiency in mice lacking the major receptor subunit. Its obligate requirement for the stable expression of oligomeric receptor. J Biol Chem. 2001;276(16):12624-8. doi: 10.1074/jbc.M011063200. PubMed PMID: 11278827.

18. Jin L, Tang Q, Hu S, Chen Z, Zhou X, Zeng B, et al. A pig BodyMap transcriptome reveals diverse tissue physiologies and evolutionary dynamics of transcription. Nat Commun. 2021;12(1):3715. doi: 10.1038/s41467-021-23560-8. PubMed PMID: 34140474; PubMed Central PMCID: PMCPMC8211698.

19. Koenig D, Jimenez-Gomez JM, Kimura S, Fulop D, Chitwood DH, Headland LR, et al. Comparative transcriptomics reveals patterns of selection in domesticated and wild tomato. Proc Natl Acad Sci U S A. 2013;110(28):E2655-62. doi: 10.1073/pnas.1309606110. PubMed PMID: 23803858; PubMed Central PMCID: PMCPMC3710864.
