# Supplementary figures and images for: Deficiency of ASGR1 in pigs recapitulates reduced risk factor for cardiovascular disease in humans
Source: PLoS Genet. 2021 Nov 11;17(11):e1009891. doi: 10.1371/journal.pgen.1009891 (PMC8584755; doi:10.1371/journal.pgen.1009891)

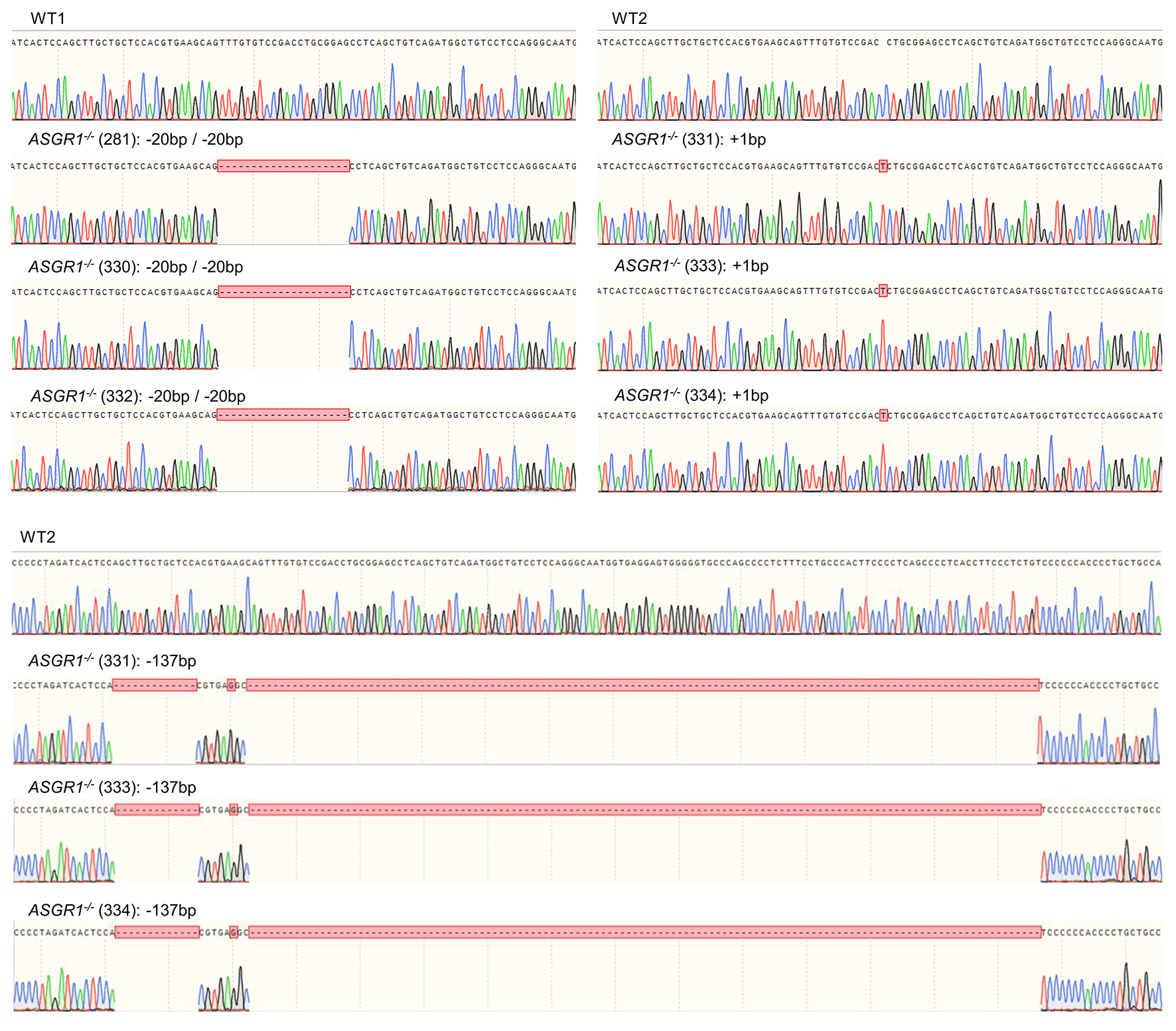

Supplement: S1 Fig — Sequencing analysis revealed that three piglets (281,330,332) carried a 20 bp deletion (-20 bp / -20 bp), corresponding to donor A1 cells. Other three piglets (331,333,334) carried a 137bp deletion and 1 bp insertion (-137 bp /+1 bp), corresponding to donor A2 cells. (TIF) [file pgen.1009891.s002.tif]

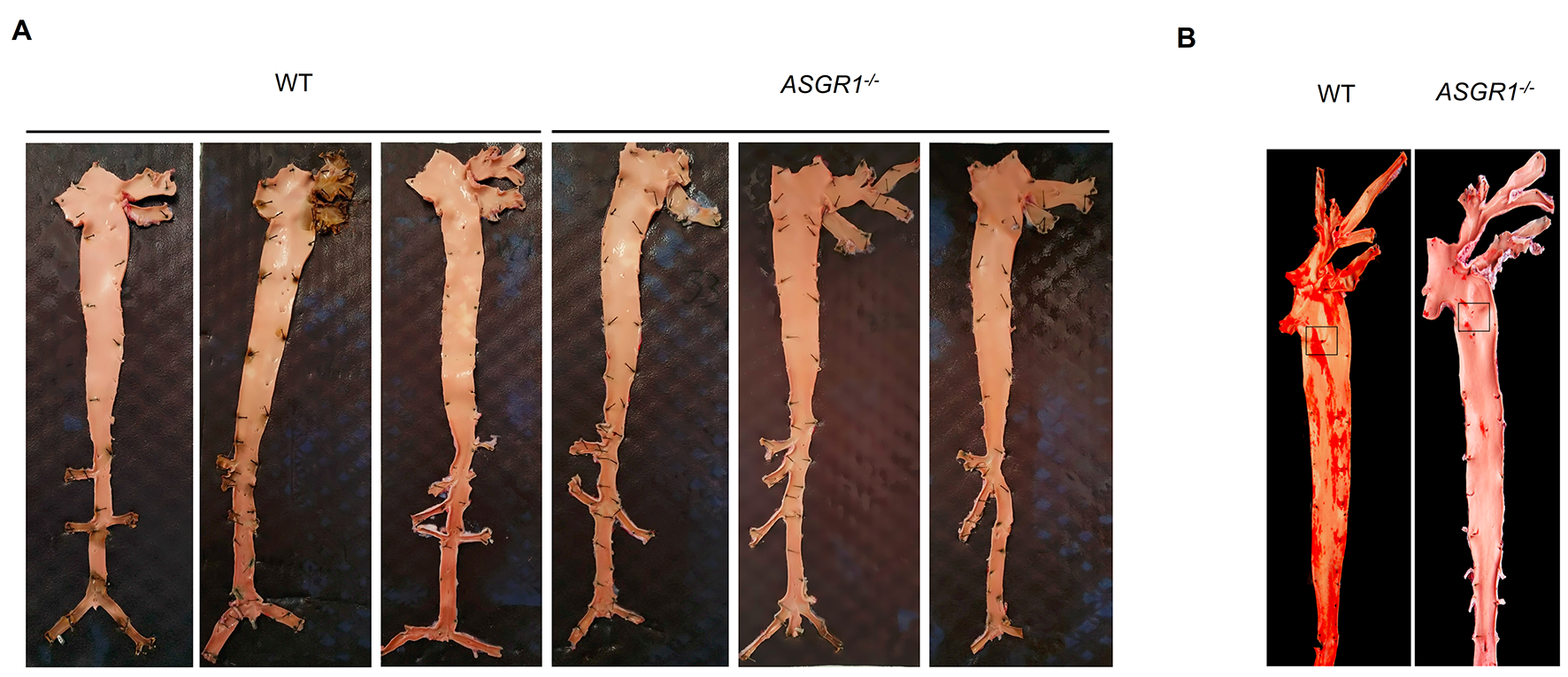

Supplement: S2 Fig — A. Representative images of Sudan IV-stained aortas of 24-month-old ASGR1-/- founder pigs and age-matched WT controls fed a normal diet. B. Representative images of Sudan IV-stained aortic arch and thoracic aorta of ASGR1+/- pigs and WT controls fed an HFHC diet. The area indicated by the black box is the area of the tissue used for histological assessment. HFHC, high-fat and high-cholesterol; WT, wild-type. (TIF) [file pgen.1009891.s003.tif]

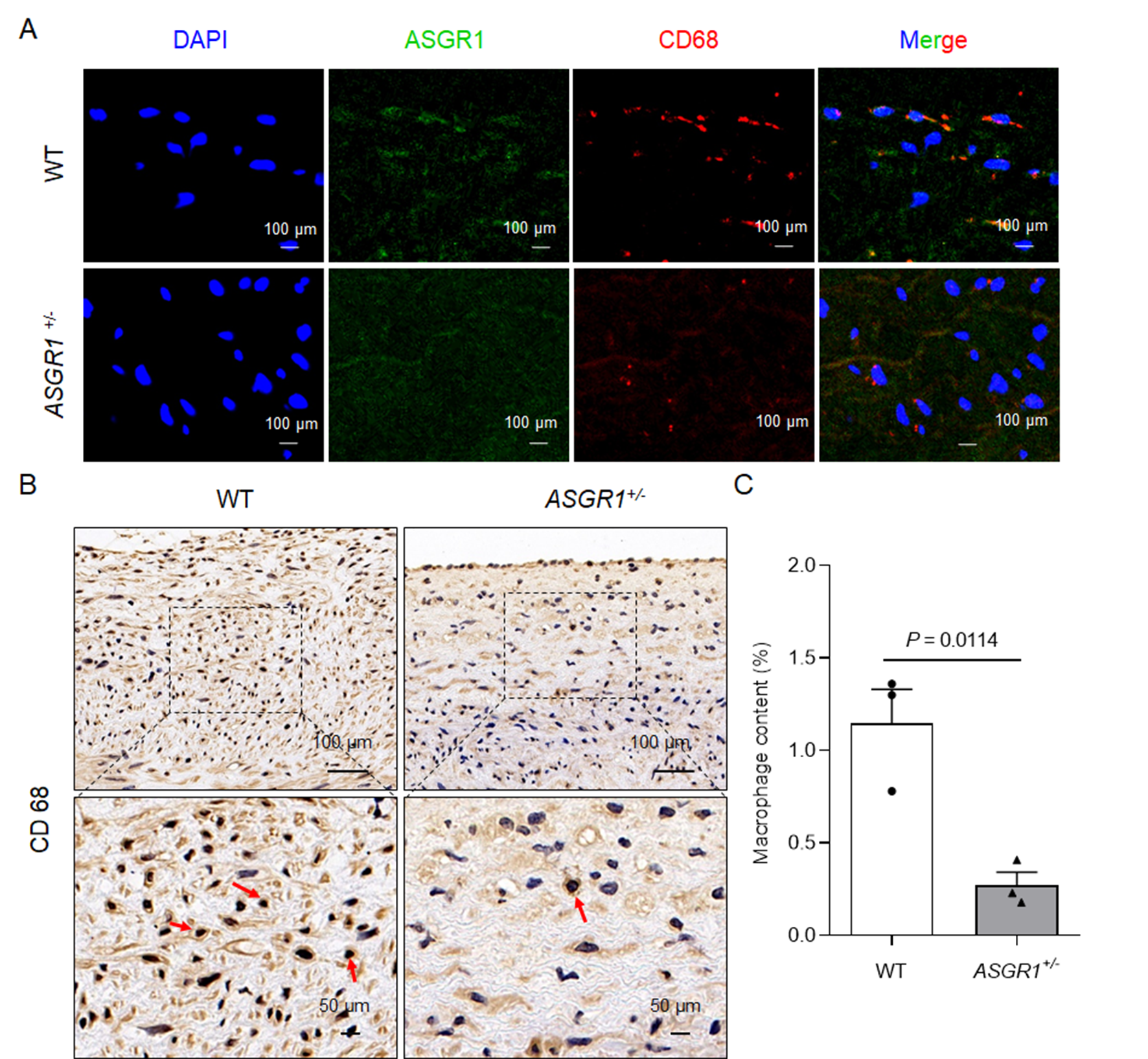

Supplement: S3 Fig — Six-month-old ASGR1+/- and age-matched WT pigs were fed an HFHC diet for six months. A. Immunofluorescence staining of CD68 in plaque from aortic arch of ASGR1+/- and WT pigs. Scale bars, 100 μm. B, C. (B) Immunohistochemistry staining and (C) quantification of CD68 of plaque from aortic arch in ASGR1+/- and WT pigs (n = 3 per group). Scale bars, 50 μm, 100 μm. Statistical analysis was performed using the Student’s t-test (P values are shown). Error bars show mean ± SEM. Points indicate data from individual pigs. The underlying data for this figure can be found in S1 Data. CD68, Cluster of Differentiation 68; HFHC, high-fat and high-cholesterol; WT, wild-type. (TIF) [file pgen.1009891.s004.tif]

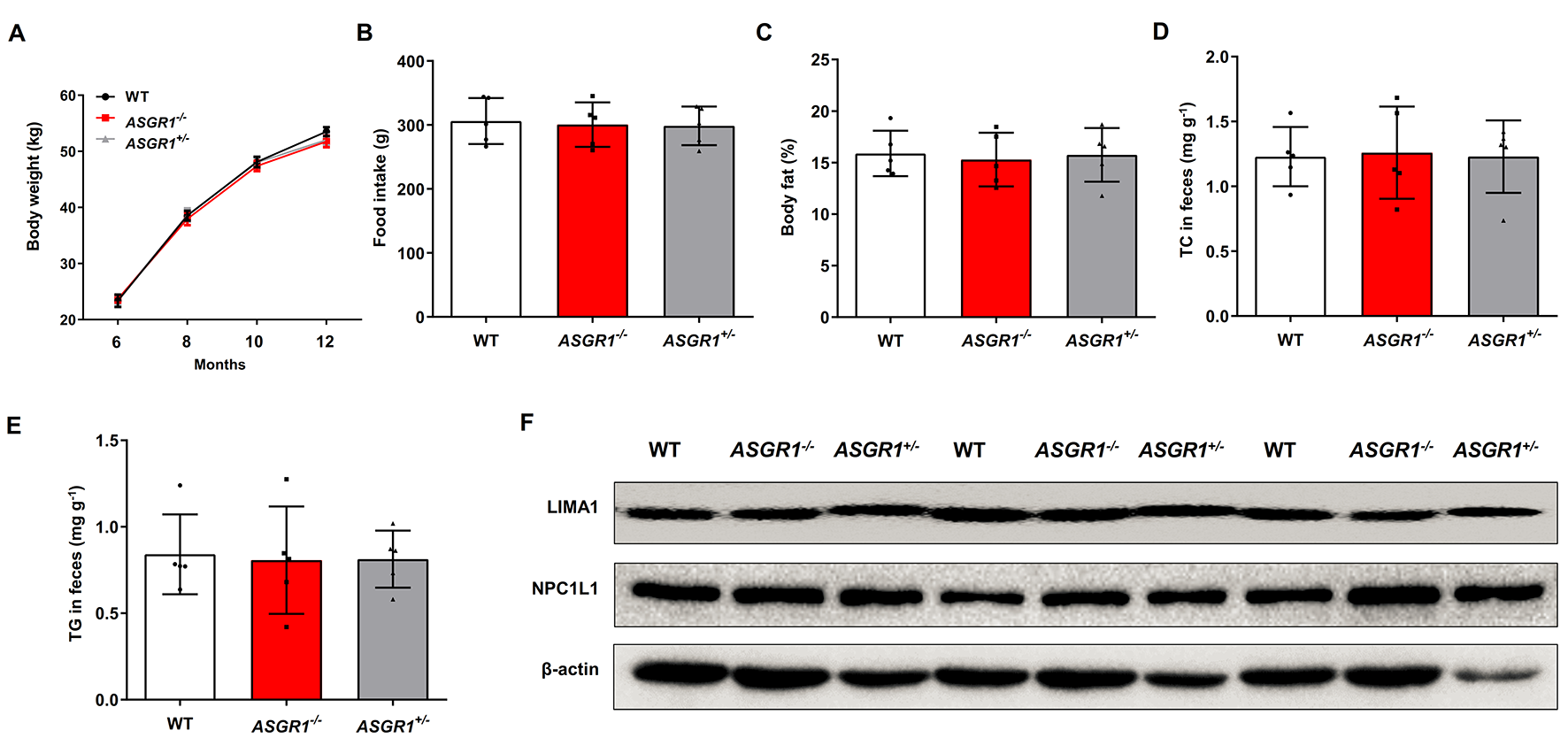

Supplement: S4 Fig — A-C. Six-month-old ASGR1-deficient pigs and age-matched WT controls fed a standard diet. (A) Body weights at 6,8,10 and 12 months. (B) Average food intake from 6 months to 12 months. (C) Body fat content at 24 months. D, E. Fecal content of (D) TC and (E) TG at 12months (n = 5 per group, data represent two independent experiments combined). F. Western blot of LIMA, NPC1L1 and β-actin (internal control) in the small intestine of 24-month-old ASGR1-deficient pigs and age-matched WT controls under normal diet. Statistical analysis was performed using the Student’s t-test (P values are shown). Error bars show mean ± SEM. Points indicate data from individual pigs. The underlying data for this figure can be found in S1 Data. LIMA1, LIM domain and actin-binding 1; NPC1L1, Niemann-Pick C1-Like 1; TC, total cholesterol; TG, triglycerides; WT, wild-type. (TIF) [file pgen.1009891.s005.tif]

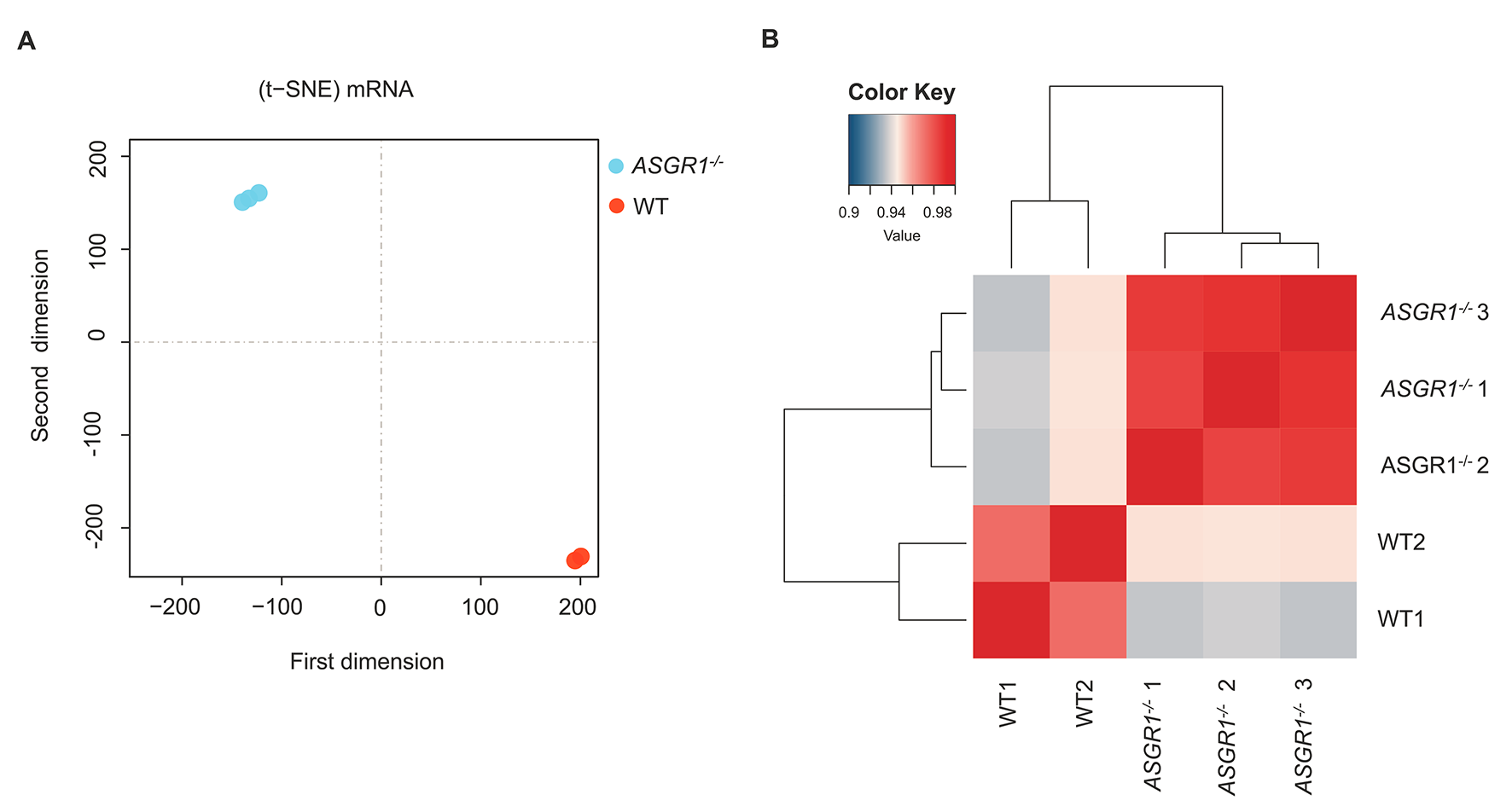

Supplement: S5 Fig — A, B. Hepatic transcriptome profiling of three ASGR-/- pigs and two WT controls. (A) Two-way t-SNE plot and (B) average linkage hierarchical clustering plot with Pearson’s correlation matrix based on the expression levels of mRNA. WT, wild-type. (TIF) [file pgen.1009891.s006.tif]

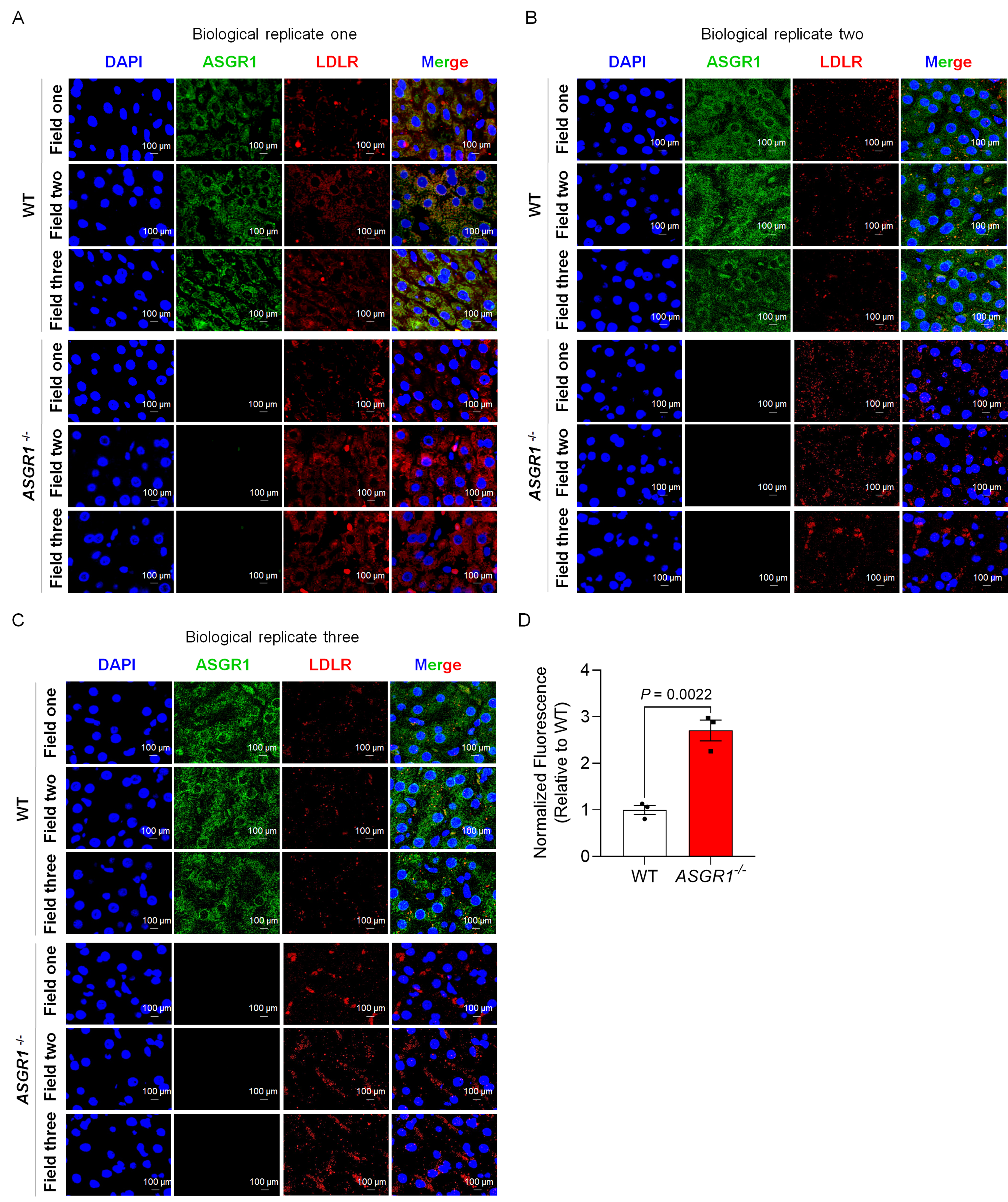

Supplement: S6 Fig — Representative immunofluorescence images of ASGR1 (green) and LDLR (red) in livers of ASGR1-/- and WT pigs. A-C. The three different fields of the three biological replicates from two independent experiments. Scale bars, 100 μm. D. The quantification of LDLR fluorescence intensities in livers of ASGR1-/- and WT pigs normalized by fluorescence values of three WT pigs (n = 3 per group, data are representative of two independent experiments). Statistical analysis was performed using the Student’s t-test (P values are shown). Error bars show mean ± SEM. Points indicate data from individual pigs. The underlying data for this figure can be found in S1 Data. ASGR1, asialoglycoprotein receptor 1; LDLR, low-density lipoprotein receptor; WT, wild-type. (TIF) [file pgen.1009891.s007.tif]

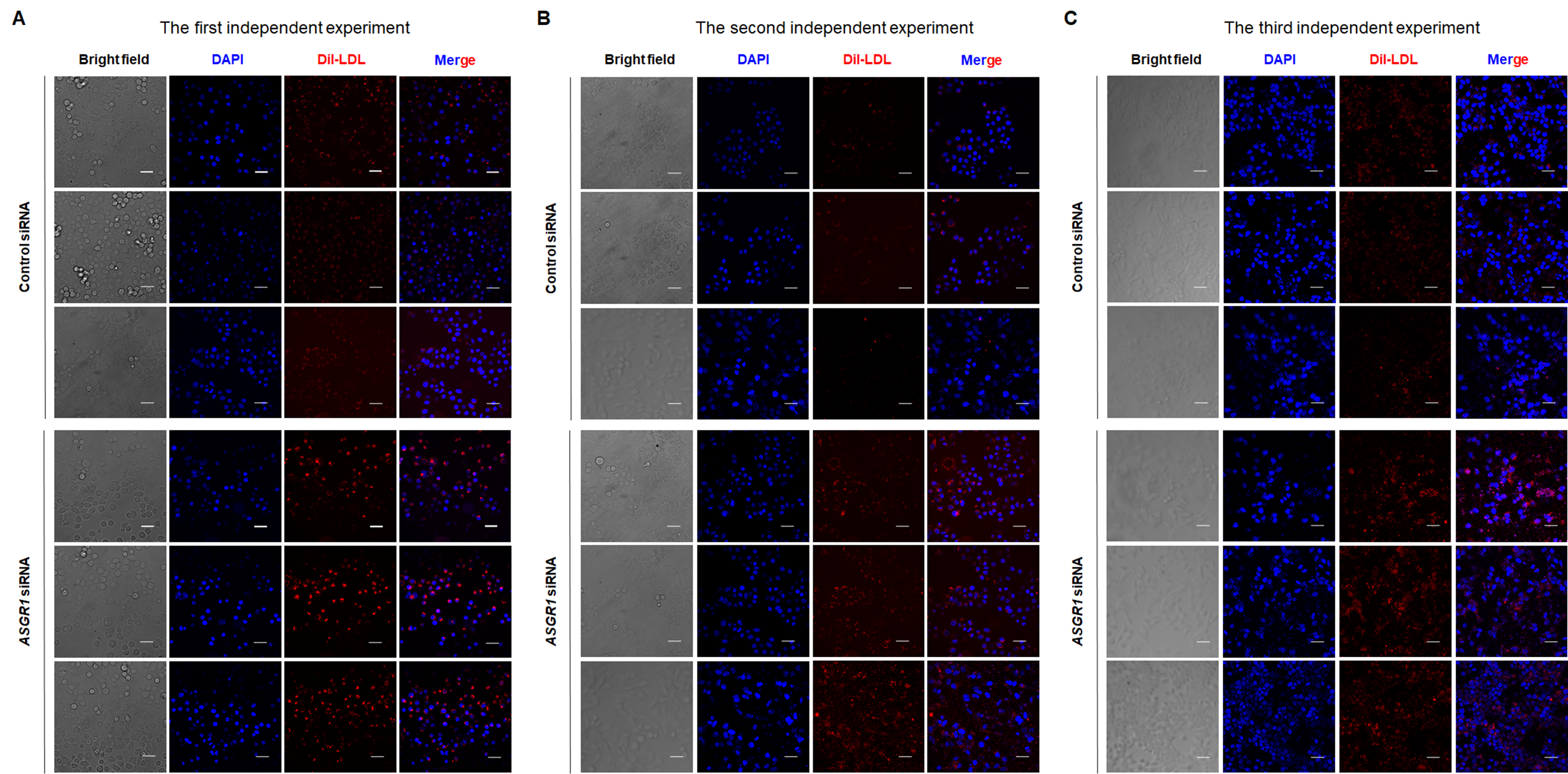

Supplement: S7 Fig — A-C. The images from the three independent experiments. Confocal microscopic images represent the fluorescence intensity of Dil-LDL (red) and DAPI (blue). Scale bars, 100 μm. DAPI, 4′, 6-diamidino-2-phenylindole. (TIF) [file pgen.1009891.s008.tif]

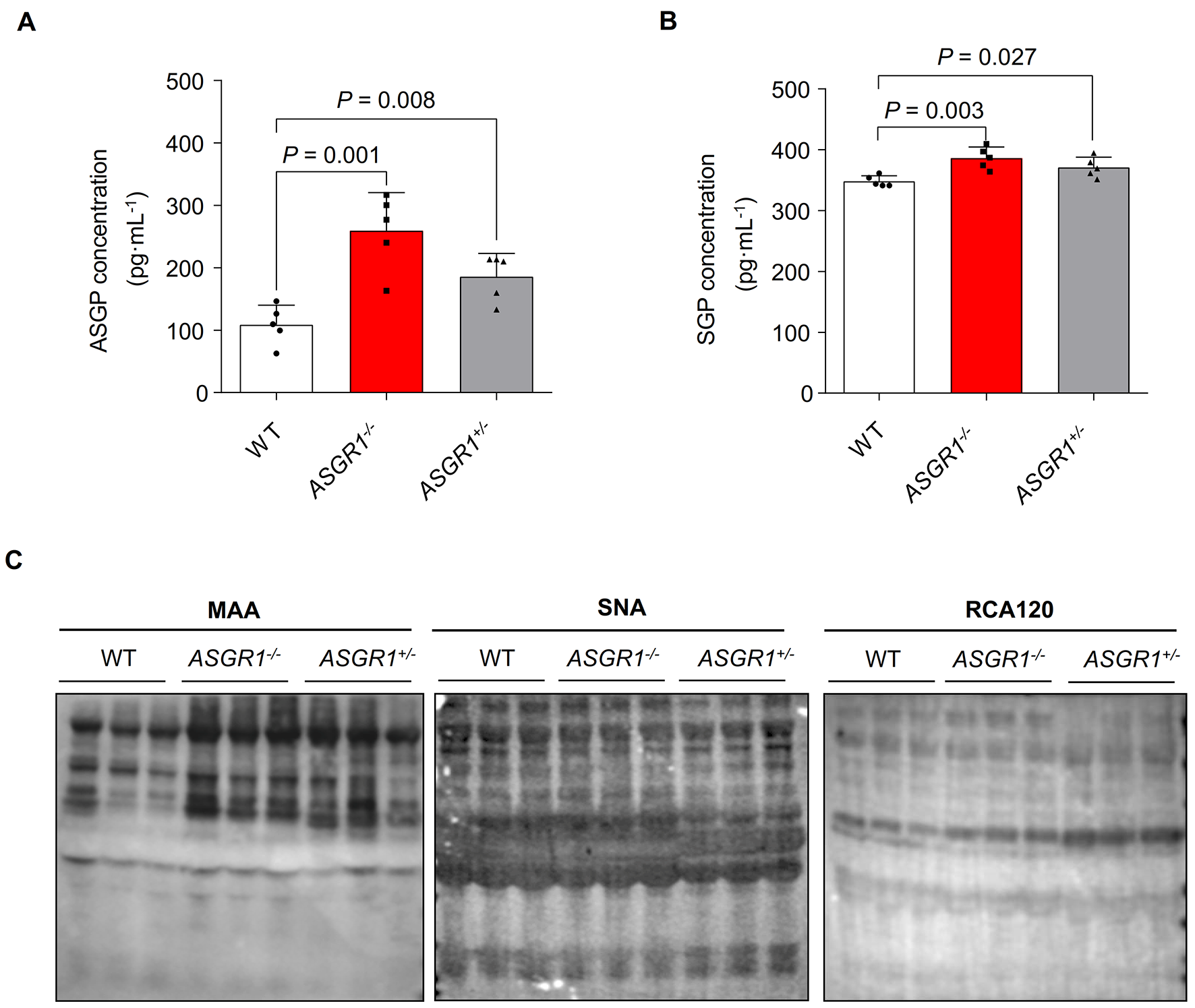

Supplement: S8 Fig — A, B. Serum levels of (A) asialoglycoprotein and (B) sialoglycoprotein were measured by ELISA in 12-month-old pigs fed a chow diet (n = 5 per group, data represent two independent experiments combined). C. Lectin blots of plasma proteins. Blood was collected from 12-month-old WT or ASGR1-deficient pigs. Lectin blots of plasma proteins using MAA, SNA and RCA120 conjugated with biotin. Biotin conjugates were respectively detected by HRP-labeled anti-biotin antibodies. Statistical analysis was performed using the Student’s t-test (P values are shown). Error bars show mean ± SEM. Points indicate data from individual pigs. The underlying data for this figure can be found in S1 Data. MAA, Maackia amurensis agglutinin; RCA120, Ricinus communis agglutinin 120; SNA, Sambucus nigra agglutinin; SNP, single nucleotide polymorphism; WT, wild-type. (TIF) [file pgen.1009891.s009.tif]

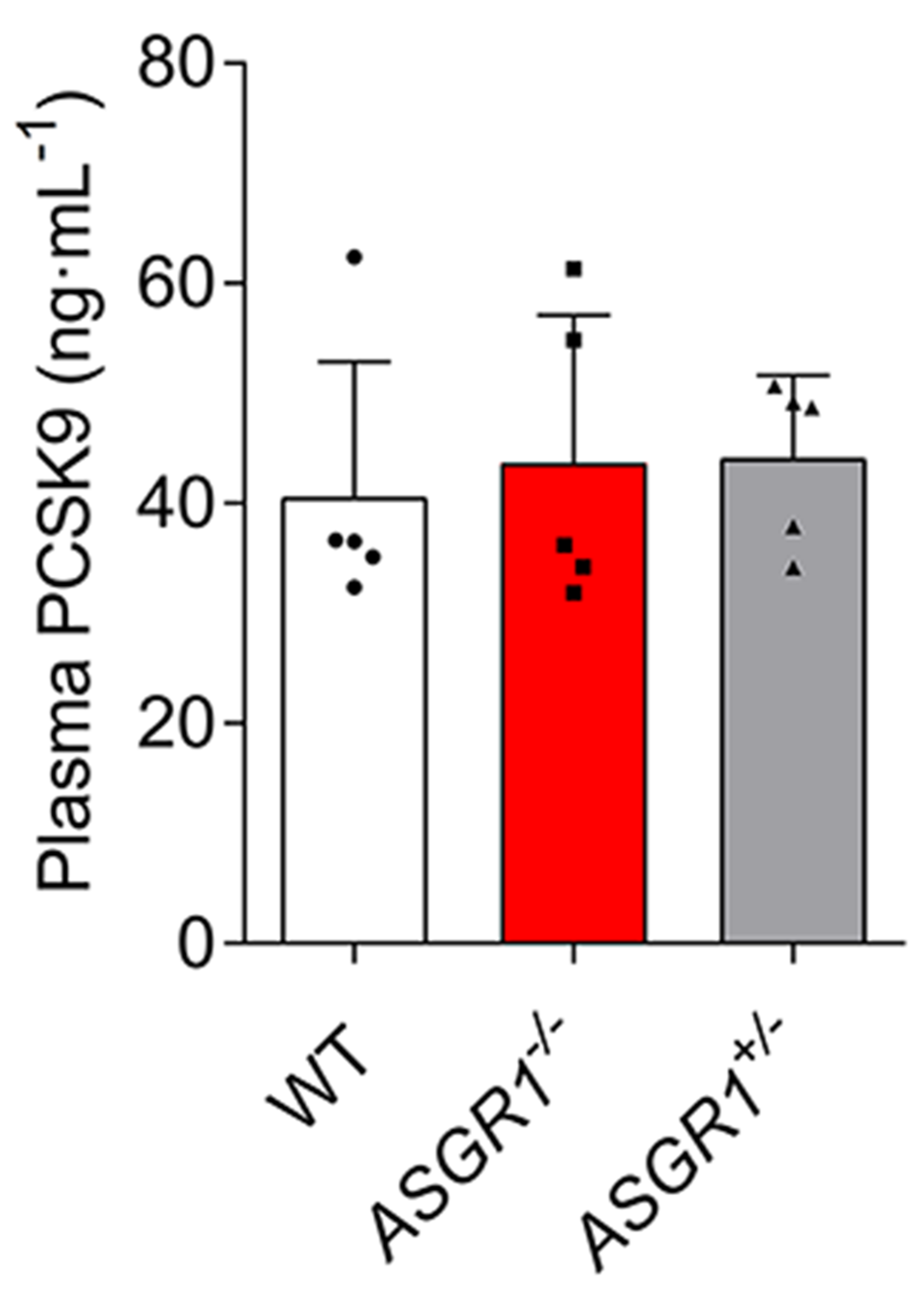

Supplement: S9 Fig — Plasma levels of PCSK9 was measured by ELISA in 12-month-old pigs fed a standard diet (n = 5 per group, data represent two independent experiments combined). Statistical analysis was performed using the Student’s t-test (P values are shown). Error bars show mean ± SEM. Points indicate data from individual pigs. The underlying data for this figure can be found in S1 Data. PCSK9, proprotein convertase subtilisin/kexin type 9; WT, wild-type. (TIF) [file pgen.1009891.s010.tif]

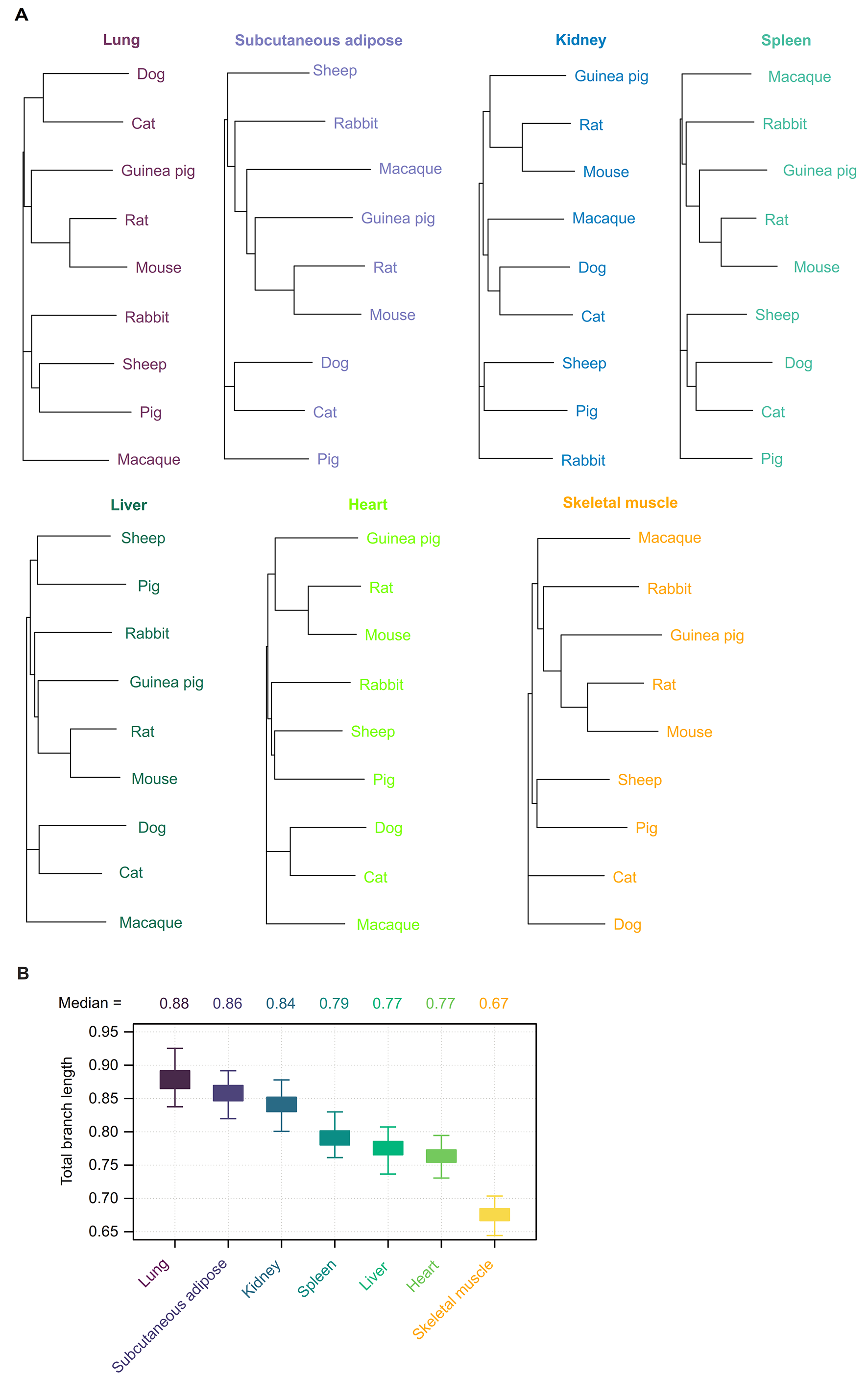

Supplement: S10 Fig — A. Gene expression phylogenies for seven tissues/organs across nine mammalian species. A neighbor-joining expression tree was constructed based on 1-Spearman correlation distances of 3,602 single-copy orthologous genes identified in all of the nine mammals. B. Box plot depicting the total branch lengths of neighbor-joining expression trees (as shown in S10A Fig) constructed based on pairwise (1-r) (r: Spearman’s correlation coefficient) distances across nine mammals for each tissue/organ. The lines inside denote the median value. (TIF) [file pgen.1009891.s011.tif]

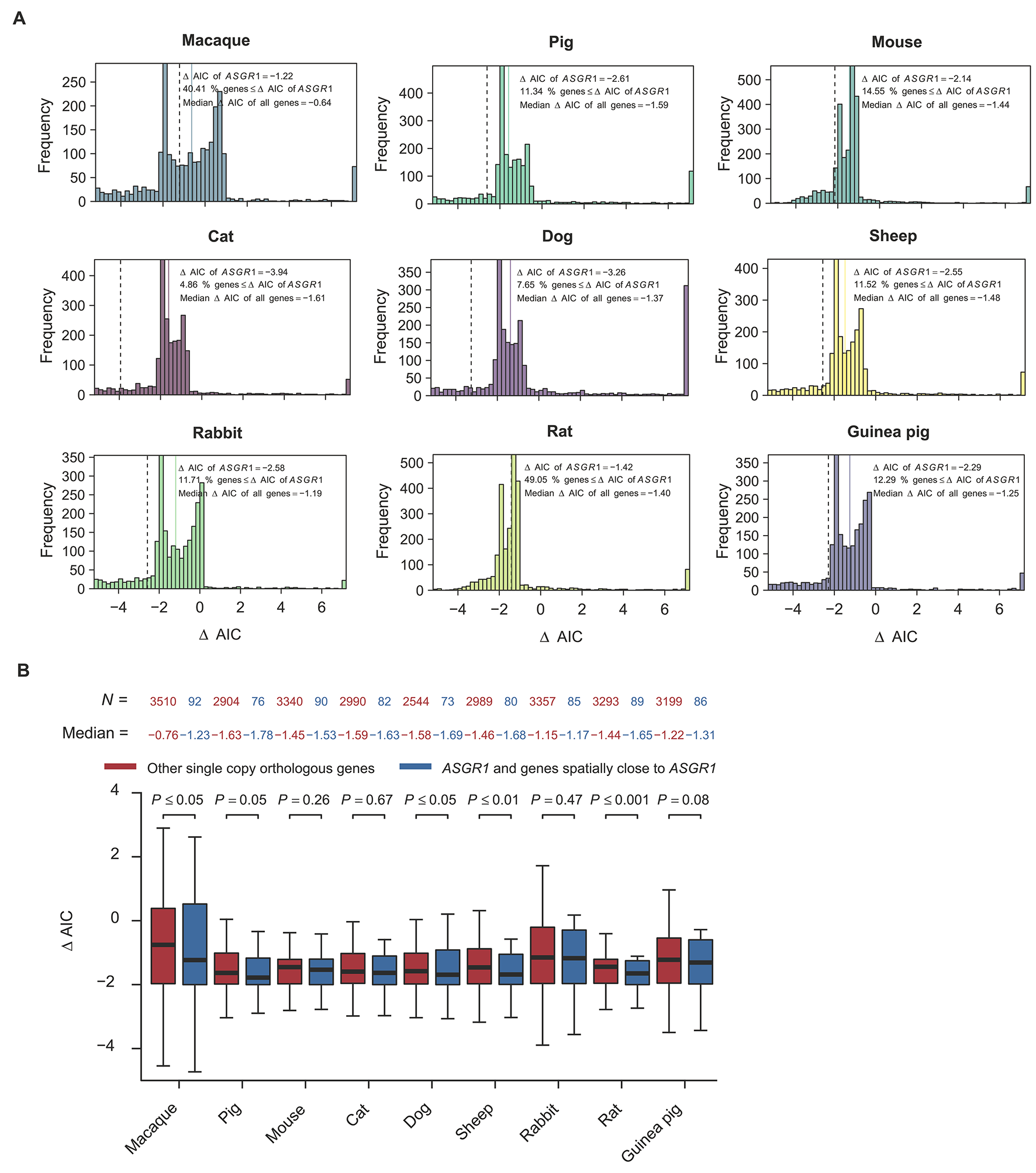

Supplement: S11 Fig — A. Histograms representing the distribution of ΔAIC values. The black vertical dashed lines indicate the ΔAIC value of ASGR1. B. Box plot depicting the distribution of ΔAIC values of genes that are spatially close to (blue) and distant from (red) ASGR1 in the liver across nine mammals. To eliminate bias effects when comparing two populations with large magnitude differences, we randomly selected non-ASGR1 single copy orthologous genes that are spatially close to ASGR1 and calculated the median ΔAIC value. We repeated this process 10,000 times. The Wilcoxon rank sum test was then performed to compare ΔAIC values between ASGR1 and its spatially close genes, and randomly generated median values. (TIF) [file pgen.1009891.s012.tif]

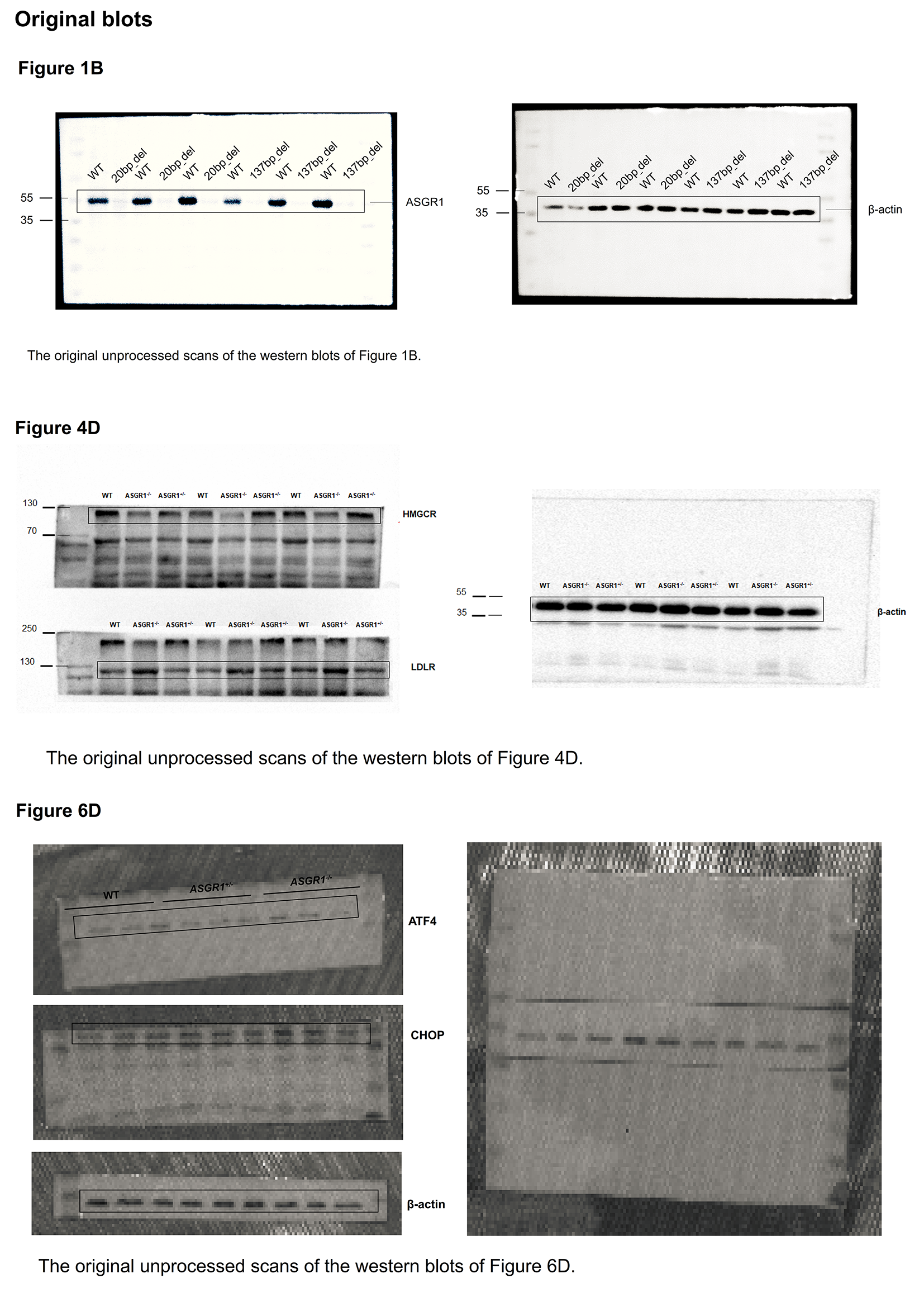

Supplement: S12 Fig — (TIF) [file pgen.1009891.s013.tif]
