# Supplementary material for: Deficiency of ASGR1 in pigs recapitulates reduced risk factor for cardiovascular disease in humans
Source: PLoS Genet. 2021 Nov 11;17(11):e1009891. doi: 10.1371/journal.pgen.1009891 (PMC8584755; doi:10.1371/journal.pgen.1009891)
Supplement: S1 Table — (DOCX) [file pgen.1009891.s014.docx]

# S1 Table Identification of *ASGR1* knockout colonies.

| **Knockout type** | **Genotype** | **Colonies** |
| --- | --- | --- |
| Homozygous | -137bp / +1bp | A2, A15 |
|  | +1bp / -20bp | A6, A7 |
|  | +1bp / +1bp | A3, A8, A10, A11, A14 |
|  | -20bp / -20bp | A1, A13 |
|  | -137bp / -137bp | A4 |
| Heterozygous | WT / -20bp | A5, A12 |
|  | WT / +1bp | A9 |
