# Supplementary material for: Deficiency of ASGR1 in pigs recapitulates reduced risk factor for cardiovascular disease in humans
Source: PLoS Genet. 2021 Nov 11;17(11):e1009891. doi: 10.1371/journal.pgen.1009891 (PMC8584755; doi:10.1371/journal.pgen.1009891)
Supplement: S2 Table — (DOCX) [file pgen.1009891.s015.docx]

# S2 Table Efficiency of SCNT in generating ASGR1-deficient pigs.

| **Recipients serial number** | **Donor cells** | **Transplant reconstructed embryos** | **Pregnancy in 28 days** | | **Litter size** | **Live-born piglets** |
| --- | --- | --- | --- | --- | --- | --- |
| 618 | A1 (-20bp/-20bp) | 123 | | + | 1 | 1 |
| 632 | A1 (-20bp/-20bp) | 50 | | + | 6 | 5 |
|  | A2 (-137bp/+1bp) | 90 | |  |  |  |
|  | A3 (+1bp/+1bp) | 98 | |  |  |  |
| Total | 3 | 361 | | 2 | 7 | 6 |
