# Supplementary material for: Deficiency of ASGR1 in pigs recapitulates reduced risk factor for cardiovascular disease in humans
Source: PLoS Genet. 2021 Nov 11;17(11):e1009891. doi: 10.1371/journal.pgen.1009891 (PMC8584755; doi:10.1371/journal.pgen.1009891)
Supplement: S3 Table — (DOCX) [file pgen.1009891.s016.docx]

# S3 Table The number of ASGR1-deficient pigs in each generation.

| **Generation** | **Number** |
| --- | --- |
| F0 | 6 |
| F1 | 15 |
| F2 | 17 |
