# Supplementary material for: Deficiency of ASGR1 in pigs recapitulates reduced risk factor for cardiovascular disease in humans
Source: PLoS Genet. 2021 Nov 11;17(11):e1009891. doi: 10.1371/journal.pgen.1009891 (PMC8584755; doi:10.1371/journal.pgen.1009891)
Supplement: S4 Table — (DOCX) [file pgen.1009891.s017.docx]

# S4 Table Results of off-target analysis.

| **Number** | **Chromosome** | **Sequence of off-target site** | **No. Mismatch** | **Off-target analysis *ASGR1* knockout piglets** | |
| --- | --- | --- | --- | --- | --- |
| 1 | chr7 | AGCCGAGTGTGTCCGACCTGCAG | 3 | | WT |
| 2 | chr10 | AGAGCTCTGTGTCCGACCTGGAG | 4 | | WT |
| 3 | chr1 | AGCTGTTTCTGTCCGACCTATGG | 3 | | WT |
| 4 | chr10 | AGCAGGTTGCATCCGACCTGAGG | 3 | | WT |
| 5 | chr3 | TTCAATTTGTGTCCGACCTCTGG | 4 | | WT |
| 6 | chr13 | CGCTGTTTGGGGCCGACCTGGAG | 4 | | WT |
| 7 | chr14 | GGAAGTTGGTGTCCGAGCTGCAG | 4 | | WT |
| 8 | chr6 | TGCAGTGTGTGTCCCACCTGGGG | 3 | | WT |
| 9 | chr8 | GGGAATTTGTGTTCGACCTGTAG | 4 | | WT |
| 10 | chr9 | ACCGCTTTGTGTGCGACCTGCAG | 4 | | WT |
