# Supplementary material for: Deficiency of ASGR1 in pigs recapitulates reduced risk factor for cardiovascular disease in humans
Source: PLoS Genet. 2021 Nov 11;17(11):e1009891. doi: 10.1371/journal.pgen.1009891 (PMC8584755; doi:10.1371/journal.pgen.1009891)
Supplement: S5 Table — (DOCX) [file pgen.1009891.s018.docx]

# S5 Table Primers used for off-target analysis.

| **Number** | **Forward prime (5'-3')** | **Reverse primer (5'-3')** | **Amplicon length (bp)** |
| --- | --- | --- | --- |
| 1 | GACGCGGTTTCCTCTTTTGT | GACTAGAAGGGAAGGAGGCC | 528 |
| 2 | ACGTGTCATTGTGCAGTTGT | GCCCCTCACAGAGTCCATTT | 555 |
| 3 | ACAAGTGATGAGACCCAAAGAA | TCTCCCCTTATGGTGGCTTT | 504 |
| 4 | CCCTTCTTTGCTGGCTTCTG | GACCAGAGCCCATGTTTGTG | 408 |
| 5 | CTGTCAGAAACCAACACGCA | GCCACTTCCCACAGCAAATG | 532 |
| 6 | TGTGTTGTAAAATCTGCCAGCA | TTTTCCCAGCGTTCCTTGTC | 514 |
| 7 | GTGCCGTTTTCTACTGTTCAGA | ACAACCAAAAGCCATCCAGC | 548 |
| 8 | GGACATCCTGAACTCACCCA | GGTTAGGTTTCGGGAGGACA | 567 |
| 9 | GGTAACAGCAGTCCCTTTTCA | TCTCTCCCAGGAAAGCTAGAG | 510 |
| 10 | ACACGAATCTCTGTCTCCGG | TTAGCTCCCACCCCTGAATG | 476 |
