# Supplementary material for: Deficiency of ASGR1 in pigs recapitulates reduced risk factor for cardiovascular disease in humans
Source: PLoS Genet. 2021 Nov 11;17(11):e1009891. doi: 10.1371/journal.pgen.1009891 (PMC8584755; doi:10.1371/journal.pgen.1009891)
Supplement: S6 Table — (DOCX) [file pgen.1009891.s019.docx]

# S6 Table Primers used for gene expression analysis.

| **Gene** | **Accession Number** | **Orientation** | **Primer Sequences (5′- 3′)** | **Production Length (bp)** |
| --- | --- | --- | --- | --- |
| *HMGCR* | [NM_001122988.1](https://www.ncbi.nlm.nih.gov/entrez/viewer.fcgi?db=nucleotide&id=172072666) | Forward | TCGTGACTGCCATCTACATTGC | 83 |
|  |  | Reverse | CGCTTCCATTAAAGTAATACAGTTGGA |  |
| *LDLR* | [NM_001206354.2](https://www.ncbi.nlm.nih.gov/entrez/viewer.fcgi?db=nucleotide&id=341604733) | Forward | CGGATTTCAAGAGCGCACAC | 74 |
|  |  | Reverse | ACTTCATGCTTCCAGCCTCC |  |
| *ATF4* | [NM_001123078.1](https://www.ncbi.nlm.nih.gov/entrez/viewer.fcgi?db=nucleotide&id=178056676) | Forward | AGTCCTTTTCTGCGAGTGGG | 80 |
|  |  | Reverse | CTGCTGCCTCTAATACGCCA |  |
| *DDIT3* | NM_001144845.1 | Forward | AGCTCTGATTGACCGGATGG | 83 |
|  |  | Reverse | AAGGTCAGCAGTAGCCCAAG |  |
| *IL-6* | [NM_001252429.1](https://www.ncbi.nlm.nih.gov/entrez/viewer.fcgi?db=nucleotide&id=356991257) | Forward | TGGATAAGCTGCAGTCACAG | 109 |
|  |  | Reverse | ATTATCCGAATGGCCCTCAG |  |
| *TNF-α* | [NM_214022.1](https://www.ncbi.nlm.nih.gov/entrez/viewer.fcgi?db=nucleotide&id=47522865) | Forward | ACAGGCCAGCTCCCTCTTAT | 102 |
|  |  | Reverse | CCTCGCCCTCCTGAATAAAT |  |
| *MCP-1* | [NM_214214.1](https://www.ncbi.nlm.nih.gov/entrez/viewer.fcgi?db=nucleotide&id=47523511) | Forward | ATCTGTGCAGAACCCAAGCA | 81 |
|  |  | Reverse | TCAAGGCTTCGGAGTTTGGT |  |
| *NOS2* | [XM_013981169.2](https://www.ncbi.nlm.nih.gov/entrez/viewer.fcgi?db=nucleotide&id=1191818853) | Forward | ACACCCCAAATACGAGTGGTTC | 129 |
|  |  | Reverse | CCCATGTACCAGCCATTGAAG |  |
| *CXCL9* | [NM_001114289.2](https://www.ncbi.nlm.nih.gov/entrez/viewer.fcgi?db=nucleotide&id=186972133) | Forward | GTATCATCTTCCTGACTCTGATTGG | 276 |
|  |  | Reverse | TCCCTTTCTTTTGCTTTTTCTTTAG |  |
| *TGF-β1* | [NM_214015.2](https://www.ncbi.nlm.nih.gov/entrez/viewer.fcgi?db=nucleotide&id=1148291168) | Forward | AGAGGGTTTTCGCCTCAGTG | 78 |
|  |  | Reverse | TTGAACCCGTTAATTTCCACG |  |
| *MMP9* | [NM_001038004.1](https://www.ncbi.nlm.nih.gov/entrez/viewer.fcgi?db=nucleotide&id=83921636) | Forward | TCTTCTGGCGTGTGAGTTCC | 73 |
|  |  | Reverse | AGGAGGTCGAAGGTCACGTA |  |
| *α-SMA* | [NM_001164650.1](https://www.ncbi.nlm.nih.gov/entrez/viewer.fcgi?db=nucleotide&id=257470978) | Forward | CAATGAGCTTCGTGTTGCCC | 88 |
|  |  | Reverse | GGTCATCTTCTCCCGGTTGG |  |
| *CTGF* | [NM_213833.2](https://www.ncbi.nlm.nih.gov/entrez/viewer.fcgi?db=nucleotide&id=1149122676) | Forward | ACCCAACTATGATGCGAGCC | 106 |
|  |  | Reverse | GCGTTGTCATTGGTAACCCG |  |
| *TIMP1* | [NM_213857.1](https://www.ncbi.nlm.nih.gov/entrez/viewer.fcgi?db=nucleotide&id=47523143) | Forward | AGCCAGGAGTTTCTCATAGC | 137 |
|  |  | Reverse | TCACAGCCAGCAGCATAG |  |
