# Supplementary material for: Deficiency of ASGR1 in pigs recapitulates reduced risk factor for cardiovascular disease in humans
Source: PLoS Genet. 2021 Nov 11;17(11):e1009891. doi: 10.1371/journal.pgen.1009891 (PMC8584755; doi:10.1371/journal.pgen.1009891)
Supplement: S7 Table — (DOCX) [file pgen.1009891.s020.docx]

# S7 Table Antibody information.

| **Antibodies** | **Source** | **Identifier** |
| --- | --- | --- |
| ASGR1 | Santa Cruz, CA, USA | sc-393849 |
| LDLR | Abcam, Cambridge, UK | ab30532 |
| HMGCR | Abcam, Cambridge, UK | ab242315 |
| LIMA1 | Abcam, Cambridge, UK | ab154530 |
| NPC1L1 | Abcam, Cambridge, UK | ab124801 |
| ATF4 | Proteintech, Chicago, IL, USA | 10835-1-AP |
| CHOP | Proteintech, Chicago, IL, USA | 15204-1-AP |
| β-actin | Cell signaling Technology, Boston, MA, USA | 4970S |
| Goat anti-rabbit | Abbkine, Hubei, China | A21020 |
| Goat anti-mouse | Abbkine, Hubei, China | A21010 |
